# Systematic *In Vivo* Characterization of Fluorescent Protein Maturation in Budding Yeast

**DOI:** 10.1021/acssynbio.1c00387

**Published:** 2022-02-18

**Authors:** Paolo Guerra, Luc-Alban Vuillemenot, Brady Rae, Valeriia Ladyhina, Andreas Milias-Argeitis

**Affiliations:** Molecular Systems Biology, Groningen Biomolecular Sciences & Biotechnology Institute, University of Groningen, 9747 AG Groningen, Netherlands

**Keywords:** fluorescent proteins, maturation time, optogenetics, EL222, mathematical modeling, budding yeast

## Abstract

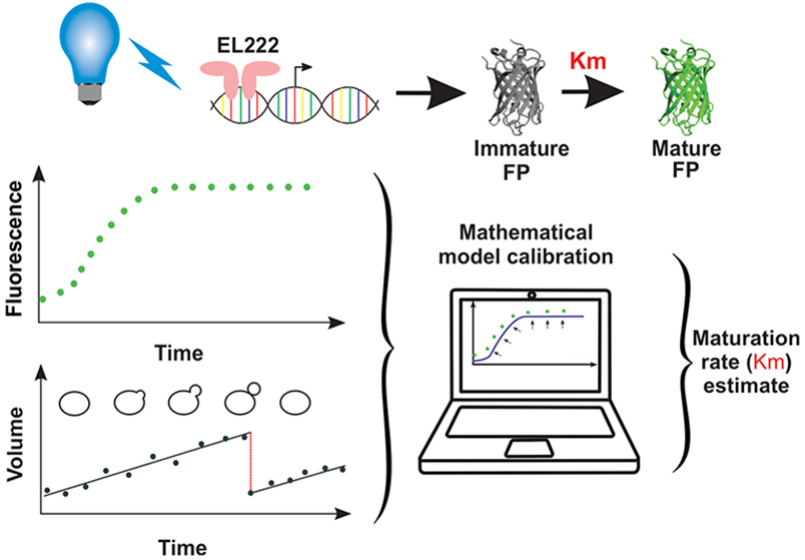

Fluorescent protein (FP) maturation can limit the accuracy with which dynamic intracellular processes are captured and reduce the *in vivo* brightness of a given FP in fast-dividing cells. The knowledge of maturation timescales can therefore help users determine the appropriate FP for each application. However, *in vivo* maturation rates can greatly deviate from *in vitro* estimates that are mostly available. In this work, we present the first systematic study of *in vivo* maturation for 12 FPs in budding yeast. To overcome the technical limitations of translation inhibitors commonly used to study FP maturation, we implemented a new approach based on the optogenetic stimulations of FP expression in cells grown under constant nutrient conditions. Combining the rapid and orthogonal induction of FP transcription with a mathematical model of expression and maturation allowed us to accurately estimate maturation rates from microscopy data in a minimally invasive manner. Besides providing a useful resource for the budding yeast community, we present a new joint experimental and computational approach for characterizing FP maturation, which is applicable to a wide range of organisms.

## Introduction

Fluorescent proteins (FPs) have become indispensable tools for the study of cellular dynamics in a wide range of applications, such as monitoring nutrient and stress responses, the quantification of gene expression noise, the measurement of protein turnover, and the characterization of synthetic inducible systems. However, FPs are not fluorescent immediately after translation. Instead, they need to undergo a process of maturation, which collectively refers to the folding and post-translational modifications that result in the formation of a functional chromophore.^1,2^ Maturation is largely autocatalytic (except for the strict requirement of molecular oxygen), but its kinetics is affected by environmental factors such as temperature.^3^ Currently available FPs have *in vivo* maturation times that range from a few minutes to hours. This fact needs to be taken into account when choosing an FP for a particular application, as the speed of FP maturation determines the range of timescales over which expression dynamics can be accurately captured.^4,5^ Moreover, slow-maturing FPs can generate artifacts in the dynamic measurements of signaling activity *via* fluorescence resonance energy transfer (FRET) biosensors.^6−8^ Besides limiting the accuracy of dynamic measurements, maturation also has a large effect on the *in vivo* brightness of a given FP.^9,10^ This is because a fraction of immature FPs is always present in dividing cell populations where FPs are continuously produced and diluted by cell growth. The size of this immature FP fraction depends on both the FP maturation rate and the cell division rate, and therefore becomes important for fast-dividing cells such as bacteria and yeast. For all these reasons, knowing the maturation rates of different available FPs is crucial for choosing the right protein for a particular application or for post-processing fluorescence time series to account for the effects of FP maturation.

Both *in vitro* and *in vivo* techniques have been developed to study the maturation kinetics. *In vitro* methods^11−13^ can provide valuable mechanistic insights under well-defined conditions. However, the intracellular biochemical environment may differ significantly from the *in vitro* environment (*e.g.,* in terms of oxygen availability, crowding, and the presence of chaperones), and these differences can lead to large discrepancies between the *in vitro* and *in vivo* folding and the maturation properties of a given FP.^9,14−18^ On the other hand, the *in vivo* assessment of FP maturation is typically carried out *via* the chemical inhibition of translation^6,9,19−22^ (sometimes combined with photobleaching of mature FPs^23^) and the quantification of the resulting fluorescence increase as immature precursors become fluorescent. However, the prolonged exposure to translation inhibitors can induce cellular stress responses and thus perturb intracellular variables that are important for FP brightness, such as pH.^14,24^ These perturbations may then affect the observed fluorescence dynamics, especially over the long timescales of slow-maturing FPs. To circumvent the potential artifacts of translational inhibition, the FP of interest has been placed under the control of a nutrient-inducible promoter. By fitting of a mathematical model of FP expression and maturation to fluorescence measurements obtained after the induction of FP transcription,^25^ an estimate of the FP maturation rate could be obtained. The main challenge of this approach is the low temporal accuracy of nutrient-based induction, which can limit the overall accuracy of the estimated parameters.

A further complication of *in vivo* maturation analysis arises from the fact that different organisms differ in terms of their intracellular biochemical environment and growth temperatures. For this reason, results from one organism may not be applicable to others, and the systematic *in vivo* characterization of FP maturation has to be carried out specifically for a given organism of interest. Systematic *in vivo* maturation studies have already been presented for bacterial^9^ and mammalian^26^ cells, with an optimal growth temperature of 37 °C. However, with the exception of a few small-scale studies,^20,23,25^ a systematic analysis of *in vivo* FP maturation in budding yeast (*Saccharomyces cerevisiae*) is still missing, despite the central importance of this model eukaryote in systems and synthetic biology. Moreover, the fact that budding yeast grows optimally at 30 °C makes it difficult to extrapolate FP maturation rates from measurements made at 37 °C.

Here, we carry out the first systematic study of *in vivo* FP maturation in budding yeast, and present the maturation rates for 12 commonly used and codon-optimized FPs. Avoiding the use of invasive techniques such as translation inhibition, we implemented a new approach that combines the optogenetic induction of FP expression with time-lapse fluorescence microscopy and mathematical modeling to estimate the FP maturation rate. Optogenetic stimulation based on the EL222 gene expression system^27,28^ provided minimally invasive, rapid, and orthogonal induction of FP transcription, while the microscopic observation of single cells growing under constant nutrient conditions enabled us to properly account for the distinctive volume dynamics of this organism, which is driven by asymmetric division. Finally, the single-cell measurements of fluorescence and growth dynamics allowed us to calibrate mathematical models describing FP expression and maturation dynamics, pinpoint FPs with one and two kinetic steps in their maturation process, and obtain accurate maturation rate estimates.

Our results reveal a large range of maturation timescales among the tested FPs, even among the proteins of the same color. Moreover, our maturation rate estimates differ in several cases from *in vitro* measurements and *in vivo* results obtained in other organisms, highlighting the importance of studying maturation in the context of an organism of interest. We further demonstrate how maturation can affect the *in vivo* brightness of an FP expressed in fast-dividing cells and how it can distort the single-cell measurements of gene expression dynamics. Besides providing a useful resource for the budding yeast community, we believe that our new experimental approach will also be applicable to other organisms thanks to the widespread availability of optogenetic gene expression systems.^29^

## Results

### Light-Inducible Expression of a Set of FPs in Budding Yeast

**Figure 1:**
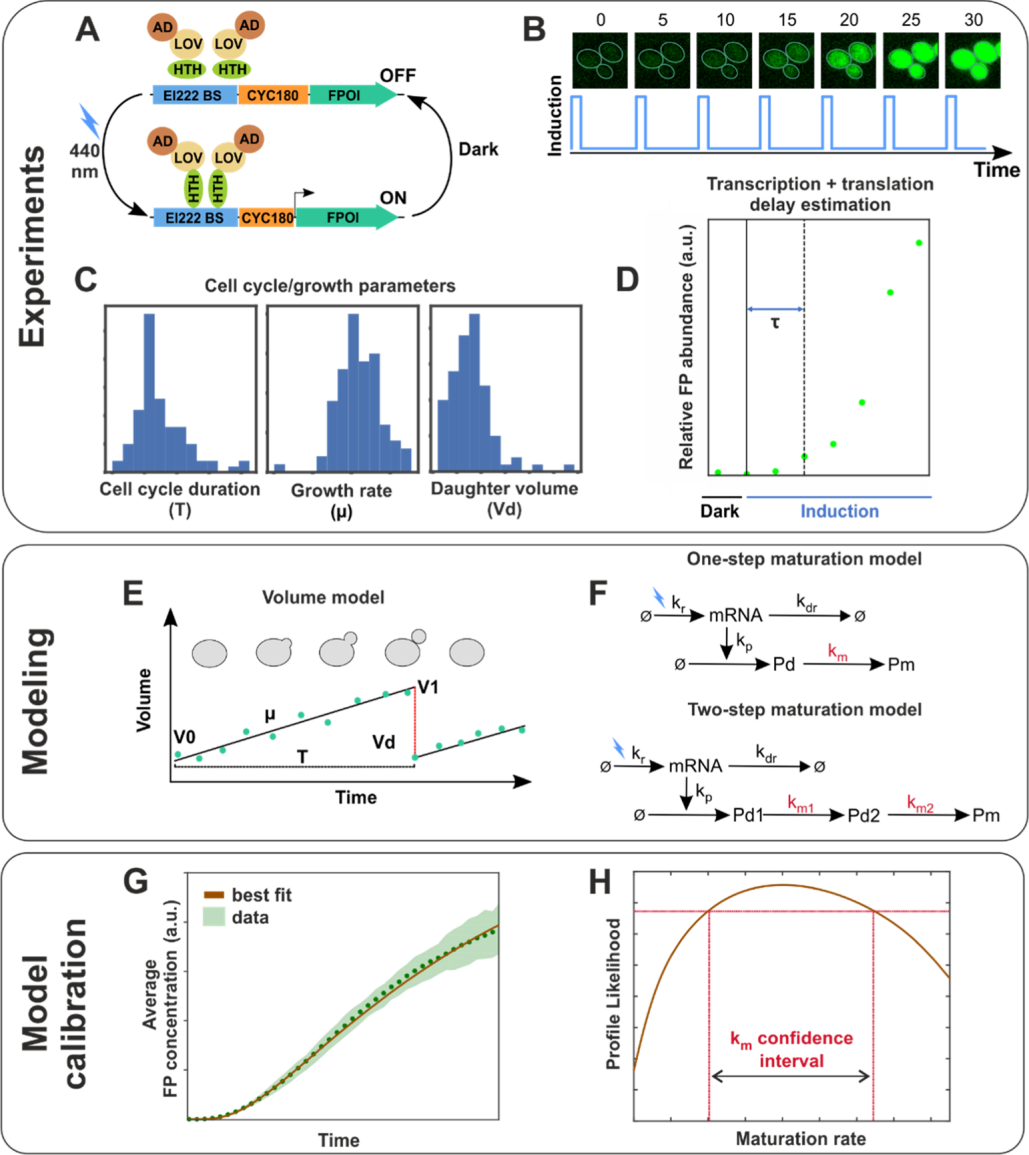
Main elements of our joint experimental/computational approach to maturation rate estimation. (A) EL222-based optogenetic gene expression system is used to drive FP expression. LOV: light–oxygen–voltage-sensing domain; AD: activation domain (VP16); HTH: helix-turn-helix DNA-binding domain; BS: binding sites; CYC180: truncated CYC1 promoter;^27^ and FPOI: FP of interest. (B) Quantification of fluorescence dynamics *via* time-lapse fluorescence microscopy of a mother cell population in which the EL222-AQTrip system drives FP expression. The cells are stimulated with short light pulses every 5 min (Figure S1). (C) Determination of cell cycle- and growth-related parameter distributions of the monitored mother cells, necessary for the simulation of cell volume dynamics [panel (E) and Figure S4]. (D) Estimation of the total delay between the activation of EL222 and the appearance of FP molecules *via* Western blotting (Figures S2 and S3). (E) Modeling the volume dynamics of a mother cell and its growing bud during a cell cycle, defined as the interval between two cytokinesis events. The continuous black line represents the linear approximation used in our model, in which a cell starts with a volume *V*_0_, grows linearly with an average growth rate μ over the cell cycle duration Τ, reaches a volume *V*_1_ at the end of the cell cycle, and loses a volume *V*_d_ (equal to the volume of the daughter cell) at division. A detailed description is provided in Methods. (F) Schematic representation of the mathematical models describing mRNA, dark protein (*P*_d_), and FP (*P*_m_) abundance assuming one-step and two-step maturation kinetics. The models are described by a set of delay differential equations, which take into account the delay between the activation of EL222 and the appearance of FP molecules [*cf.* panel (D)]. The rates of the maturation steps are denoted in red. The combination of the abundance and volume models enables for the calculation of the FP concentration over time (*cf.*Methods for further details). (G) Population-averaged fluorescence concentration of simulated cells (brown line) is fitted to the measured population-averaged fluorescence of real cells (green dots) in order to estimate the abundance model parameters, including the maturation rate *k*_m_. (H) Confidence intervals for maturation rates (and the corresponding maturation half-times) are obtained *via* the profile likelihood^35^ (Methods).

To overcome the technical challenges associated with nutrient- and chemically induced gene expression systems, we used a single-component optogenetic gene expression system based on the bacterial light–oxygen–voltage (LOV) protein EL222^27,28^ to activate the expression of FPs in budding yeast. We constructed a collection of 12 yeast strains, each carrying a chromosomally integrated copy of a slow-reverting EL222 mutant (AQTrip^30^) and a copy of a codon-optimized FP gene driven by an EL222-responsive promoter (Figure 1A and Methods). The AQTrip mutant was chosen because of its slow dark reversion (with a half-life of ∼30 min^30^), which allows the use of sparsely spaced light pulses for induction, thus avoiding potential phototoxicity effects caused by continuous illumination over long time spans (Figures 1B, S1 and Supporting Information Note 1). Because AQTrip is activated within a few seconds upon light induction, similarly to the wild-type protein,^30^ the induction of FP expression can be precisely timed, a feature that is important for precisely capturing the fluorescence dynamics *via* mathematical modeling.

For our tests, we chose a set of FPs of different colors that have been previously used in yeast, for example, in the construction of FRET-based biosensors or for monitoring dynamic changes in protein expression. To monitor FP expression dynamics, we followed the fluorescence of a yeast cell population in time using time-lapse fluorescence microscopy. A constant nutrient environment was maintained throughout the experiments by growing cells inside a microfluidic device^31^ suited for long-term cell imaging.

### Mathematical Description of FP and Single-Cell Volume Dynamics

Between the induction of FP expression and the observation of fluorescence lie the processes of transcription, translation, and maturation. Maturation, in particular, has been modeled using a single- or multistep models,^25,32,33^ depending on the features of a particular FP. To estimate FP maturation rates using a mathematical model that captures the dynamics of FP expression and maturation, we constructed a system of delay differential equations (DDEs) describing mRNA transcription from the active EL222-responsive promoter, the translation of the mRNA into an immature FP precursor form, and the formation of a FP *via* one or more rate-limiting steps (Methods and Figure 1D,F). We will henceforth refer to these equations (eqs 1a–1c, or 2a–2d in Methods) as the “abundance model”.

Budding yeast populations consist of mother and daughter cells that differ in size, morphology, cell cycle duration, and growth dynamics. This fact complicates the modeling of volume dynamics of yeast populations and generates practical challenges for the automatic single-cell quantification of protein abundance, which requires the segmentation and tracking of mother cells and their growing buds. To simplify the modeling of volume dynamics and experimental data collection, we therefore determined the mean cellular fluorescence intensity (a proxy for FP concentration)^20,34^ for a fixed number of mother cells that were present from the beginning until the end of an experiment (Methods).

To describe the concentrations of the mRNA and protein species, our abundance model was augmented with a simple individual-based stochastic model for mother volume dynamics (Figure 1C,E and eqs 1c–2d in Methods). Briefly, mother cells produce buds which grow and eventually divide, taking away a fraction of the mother–bud cell contents and volume. According to our model, during a cell cycle *c*, a mother cell of initial volume *V*_0,*c*_ grows a bud at a constant (average) rate μ_*c*_ over a cell cycle with a duration Τ_*c*_, after which the bud divides. Upon division, the bud volume (*V*_d,*c*_) is lost (Figure 1E). The distributions of all volume model parameters were estimated from the same mother cells that were tracked throughout each experiment (Methods) in order to capture the small differences in growth dynamics that are typically observed across different experiments (Figure S4A–D). With these distributions, we could generate individual mother cell volume trajectories whose features matched the volume dynamics observed in our experiments (Figure S4E,F). Using the mRNA–protein abundance model together with the volume model, we could then simulate the evolution of mRNA and protein concentrations in a population of growing and dividing mother cells (Methods) and match the population-averaged FP concentration predicted by the model with the population-averaged fluorescence concentration measurements obtained from our experiments (Figure 1G and Methods).

### Model-based estimates of FP maturation rates

For all the green and yellow-green FPs derived from *Aequorea victoria* GFP (avGFP), we implemented a one-step maturation model, reflecting the fact that the final oxidation step in the chromophore maturation process is the main rate-limiting step for these FPs.^13,36^ The maturation of Cerulean (also avGFP derived) was also adequately captured using a one-step model. However, fitting mTurquoise2 and mTFP1, and all the red-emitting FPs using a one-step model produced less satisfactory fits. In those cases, a two-step model was able to capture adequately the FP maturation dynamics and was strongly supported by the Akaike information criterion (AIC)^37^ in comparison to the one-step model (Supporting Information Note 4).

To ensure that FP maturation rates can be reliably estimated from our experimental data despite the presence of additional unknown parameters in our model, we investigated the identifiability properties of our models.^38^ Given that the volume model parameterization was provided by the experimental data, we focused on the identifiability of the abundance model, whose parameters were unknown. Structural identifiability analysis of this model verified that the maturation rate for the one-step model and the individual maturation rates of the two-step models are structurally locally identifiable (Supporting Information Note 5). However, the individual maturation rates of the two-step models are in practice difficult to distinguish from each other (*i.e.*, they are practically unidentifiable) when the individual step half-times are not too different from each other (Methods and Supporting Information Note 5). It is worth noting that these observations are valid for all linear two-step maturation models used in the literature and are not specific to our model. Given the difficulties associated with estimating two distinct maturation rates for each two-step model, we estimated a single rate parameter for both the maturation steps because the error introduced by this choice is negligible when the two rates are of the same order of magnitude (Supporting Information Note 5), that is, in the case when a two-step maturation model is really necessary (when *k*_m1_ and *k*_m2_ differ by more than an order of magnitude, the smallest rate will dominate the system response and produce single-step behavior).

To estimate the unknown parameters of the abundance model, we sought for the parameter value combination that maximized a log-likelihood function formed by the sum of squared deviations between the measured and predicted population-averaged FP concentrations over time (Figure 1G and Methods), assuming that our measurements were corrupted by additive Gaussian noise generated by measurement noise and day-to-day variability (Methods). Our likelihood function considered the fluorescence data up to 200 min post-induction, an interval which was determined to be sufficient for estimating the model parameters for all the FPs considered in this work. The maximization of the likelihood function with respect to the abundance model parameters (taking into account unidentifiable combinations, see Methods) resulted in good fits for all the FP data sets (Figure S5A–L).

Following likelihood maximization, we verified the practical identifiability of the FP maturation rate and obtained approximate confidence intervals for the maturation rates of different FPs through profile likelihood estimation^35^ (Figure 1H and Methods). The resulting optimal maturation half-time estimates (given by ln(2)/*k*_m_^*^, where *k*_m_^*^ is the maximum likelihood maturation rate estimate) and their associated 95% confidence intervals are displayed in Table 1. It should be noted that in the case of a two-step maturation model, a single maturation half-time cannot be defined based on the maturation rates of the individual steps. Instead, the sum of the half-times defined by each maturation step (ln(2)/*k*_m1_^*^ + ln(2)/*k*_m2_^*^) forms a *lower bound* on the actual maturation half-time of the protein (Supporting Information Note 5). An accurate estimate of the maturation half-time (assuming that only the nonfluorescent precursor *P*_d1_ is present initially) can be obtained by simulation and is also provided in Table 1.

**Table 1:** Estimates of Maturation Half-Times for the FPs Tested in Our Experiments^a^

one-step maturation rates		
FP	*t*_50_ (min)	95% C.I.
Cyan		
Cerulean	9.7	[5.5, 13]
Green		
sfGFP	6.9	[5, 10.5]
pH-tdGFP	13.7	[10, 21]
Yellow-Green		
mVenus	20.8	[11.5, 31]
mCitrine	10.4	[8, 20]
mNeonGreen	11.6	[7.5, 20]

two-step maturation rates			
FP	*t*_1,50_ (min)/*t*_2,50_ (min)	95% C.I.	equivalent *t*_50_ (min)
Cyan			
mTurquoise2	27.5, 27.5	[18.5, 30]	66.6
mTFP1	31.6, 31.6	[28.5, 46.5]	76.5
Red			
mScarlet-I	12.9, 12.9	[11, 17.5]	31.2
mCherry	21.6, 21.6	[20, 28]	52.3
tdTomato	38.4, 38.4	[36.5, 55]	93
mKate2	53.5, 53.5	[45, 76]	129

a For proteins with a one-step maturation kinetics, the maturation half-life time is given by *t*_50_ = ln(2)/*k*_m_^*^, where *k*_m_^*^ is the maximum likelihood estimate of the maturation rate. As discussed in Supporting Information Note 5, we estimated a single rate parameter for both the maturation steps of two-step proteins, given the practical unidentifiability of the two maturation rates. The half-time of each step is given by *t*_1,50_ = *t*_2,50_ = ln(2)/*k*_m_^*^, where *k*_m_^*^ is the (common) maximum likelihood rate estimate. 95% confidence intervals for maturation rates (and therefore for maturation half-times) were obtained *via* profile likelihood (Methods). The equivalent maturation half-time for two-step FPs was obtained *via* simulation, assuming that only the first precursor is present initially.

Given that the stochastic individual-based volume model that we used above to calculate species concentrations can be computationally intensive, we finally explored the possibility of employing a simpler DDE-based model to directly compute species concentrations over time. This deterministic model contains a linear term to capture the average effect of dilution across the monitored cell population, resulting in much faster runs (Supporting Information Note 6). Although it relies on stronger simplifying assumptions than the individual-based model, this concentration model was able to produce maturation rate estimates that were, for the most part, similar to those obtained with the individual-based model. This finding demonstrates that this simpler model is a viable alternative, especially when computational resources are limited. It should be noted, however, that the calculation of the dilution rate in this model still requires the determination of single-cell volumes and division rates, as was done for the more complex volume model above (Supporting Information Note 6).

### Testing Model Predictions

**Figure 2:**
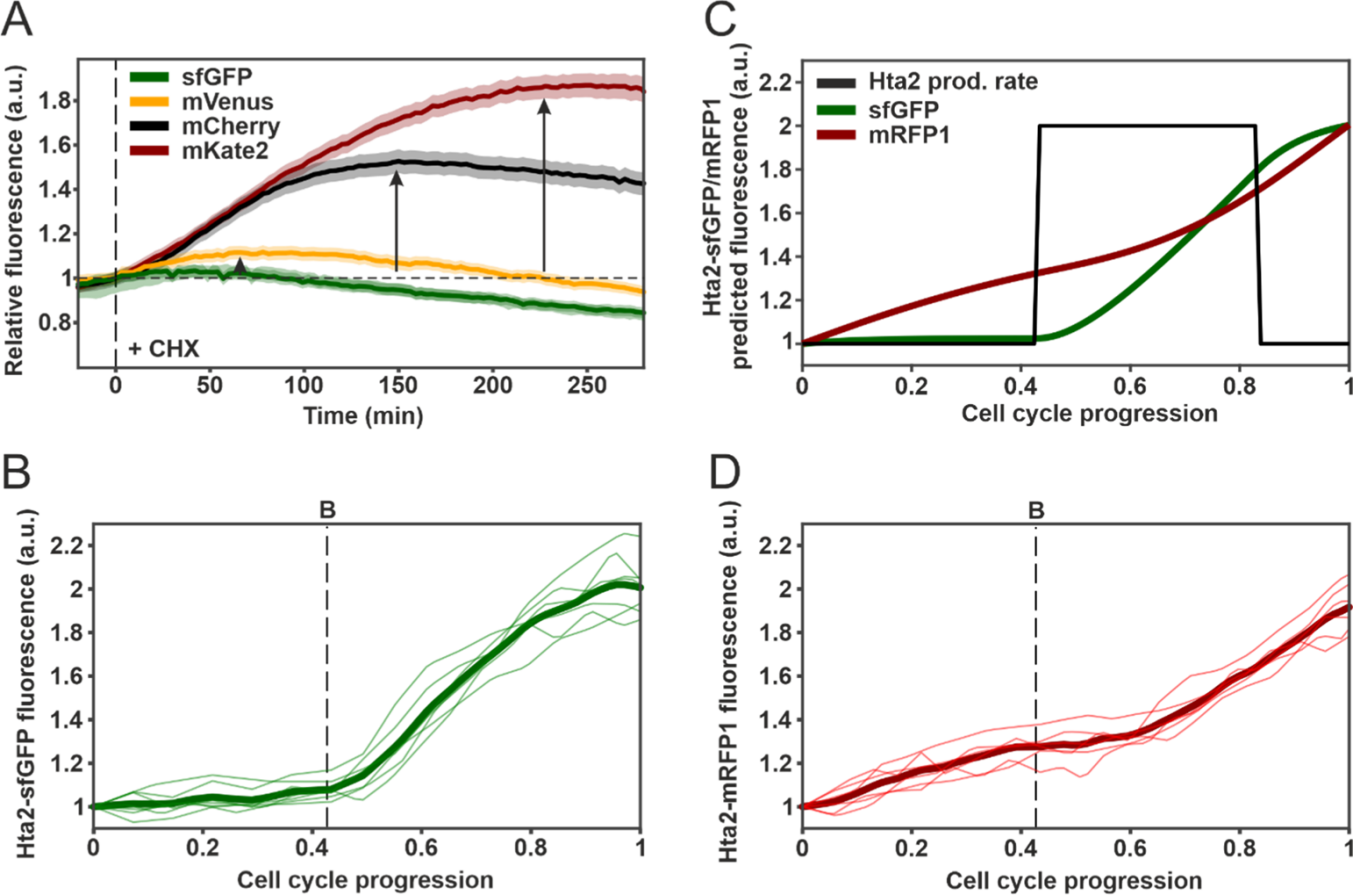
Testing model predictions. (A) Fluorescence dynamics of cells carrying sfGFP (green, *n* = 49 cells), mVenus (orange, *n* = 55), mCherry (black, *n* = 53), and mKate2 (red, *n* = 61) driven by the constitutive TEF1 promoter after the addition of CHX (25 μg/mL final) at *t* = 0 (dashed line). Fluorescence curves were normalized with respect to their value at *t* = 0 to better compare the relative increase in fluorescence. The vertical black arrows indicate the relative maximum reached by the fluorescence signal, and bands denote the standard error of the mean. The small (inconsequential) decrease in fluorescence observed at later times is not due to FP degradation (Figure S6), as we verified using Western blotting. Instead, it can be attributed to a combination of photobleaching of the FP pool, changes in the intracellular environment that may affect FP brightness, and changes in mother cell volume after CHX treatment. (B) Hta2-sfGFP single-cell fluorescence dynamics during the cell cycle (from karyokinesis to karyokinesis) (*n* = 7 cells). The dashed line denotes the moment of budding. Individual cell cycle traces were interpolated and aligned as described in Methods. The thick green line represents the mean. (C) Predicted Hta2-sfGFP (green) and Hta2-mRFP1 (red) fluorescence dynamics during the cell cycle assuming a pulsatile Hta2 production rate (black). One-step maturation kinetics and a maturation half-time of 7 min were used to model Hta2-sfGFP dynamics. A two-step model with individual maturation half-times of 22 min were used to model Hta2-mRFP1 dynamics. The time axis was normalized from 0 to 1 to represent progression through the cell cycle. Furthermore, details on the histone–FP model are provided in the Supporting Information Note 3. (D) Hta2-mRFP1 single-cell fluorescence dynamics during the cell cycle (from karyokinesis to karyokinesis) (n = 6 cells). The dashed line denotes the moment of budding. Individual cell cycle traces were interpolated and aligned as described in Methods. The thick red line represents the mean.

As a first test of our model predictions on the relative maturation rates of different FPs, we performed a protein synthesis inhibition experiment, similar to the assay used to estimate FP maturation rates *in vivo*.^6,9,19,20^ In this experiment, we added the translation inhibitor cycloheximide (CHX) to growing and dividing yeast cells expressing different FPs under the control of the constitutive TEF1 promoter. When CHX reaches the cells, it blocks the production of new FP precursors, stops cell division, and slows down volume increase. The maturation of immature FP precursors that were produced prior to CHX addition will therefore lead to an increase in the fluorescence intensity of the cells. The concentration of these immature precursors, and thus the fluorescence increase, depends on the maturation rate of the FP. Based on this reasoning, we expected that the addition of CHX to constitutively expressed FPs with different maturation rates would lead to fluorescence changes that reflect the relative maturation rates of these proteins. For fast-maturing FPs, the increase in fluorescence should be small, reflecting the fact that the immature precursor concentration is a small fraction of the total FP concentration. On the other hand, the immature fraction should be larger for slow-maturing FPs, leading to a correspondingly larger fluorescence increase after CHX addition. In line with the maturation rate estimates of Table 1, the CHX treatment of cells expressing sfGFP, mVenus, mCherry, and mKate2 resulted in fluorescence increases that were ordered in exactly the same way as the maturation half-times of these proteins (Figure 2A). These observations also demonstrate the fact that the *in vivo* brightness of a slowly maturing FP in fast-dividing cells is severely limited by the maturation kinetics because a large fraction of the expressed FP remains invisible.

Besides affecting the *in vivo* brightness, differences in FP maturation rates manifest themselves in highly dynamic settings, such as in the monitoring of cell cycle-regulated gene expression. A prime example of cell cycle-regulated proteins is histones. Because the synthesis of histones is tightly coupled to DNA replication,^39−41^ the dynamics of the fluorescently tagged core histone Hta2 can in principle be used to determine the interval of DNA replication during the budding yeast cell cycle. Given the relatively short timescale of this process (average S/G2/M duration in fast-growing budding yeast is around 60 min) and the fact that many of our FPs had comparable maturation half-times, we reasoned that the maturation rate of the FP used in the Hta2-FP fusion construct could potentially distort the observed histone dynamics during the cell cycle.

To explore this possibility, in a second test, we compared the cell cycle dynamics of Hta2-sfGFP and Hta2-mRFP1 (a precursor of mCherry with a similar reported maturation timescale^9^) by measuring the total fluorescence of cells growing on minimal glucose medium for several cell cycles (Methods). To arrive at cell cycle histone profiles, we interpolated, aligned, and averaged individual cell cycle fluorescence time series using the appearance of the bud (entry to S) and karyokinesis (the onset of anaphase) as cell cycle indicators (Methods). Following histone abundance with the fast-maturing sfGFP correctly showed a plateau during G1, where no DNA replication takes place, followed by an increase starting soon after budding and a second plateau prior to karyokinesis, correctly indicating that histone synthesis starts right after budding and stops several minutes before the anaphase (Figure 2B). A simple mathematical model that assumed a pulse for the histone synthesis rate and accounted for sfGFP maturation (Supporting Information Note 3) was able to correctly predict the observed fluorescence dynamics (Figure 2C).

In stark contrast to sfGFP, use of the slow-maturing mRFP1 as a reporter of histone synthesis produced a very different fluorescence pattern, with the signal increasing throughout the whole cell cycle, even during G1 and late G2/M when no histone synthesis occurs (Figure 2D). Assuming the same histone synthesis rate dynamics as before and accounting for mRFP1 maturation with a two-step model using the maturation rates estimated for mCherry (Supporting Information Note 3), we correctly predicted the observed fluorescence dynamics. The model also explains the “paradoxical” increase in fluorescence during G1 (when histones are not produced), which is due to the fact that a fraction of the immature mRFP1 synthesized during the previous cell cycle is still maturing during the G1 phase of the following cell cycle (Figure 2C,D).

Collectively, the tests described above showed that our maturation rate estimates are in good agreement with experimental observations. They also highlight the effect of maturation on the *in vivo* brightness of a given FP, and on the observations of dynamic single-cell gene expression patterns.

## Discussion

Maturation is an FP characteristic that is often underappreciated, even though it plays a crucial role in the studies of gene expression dynamics inside living cells and also contributes to the *in vivo* brightness of a given FP in dividing cell populations. A previous study of FP maturation in the model prokaryote *Escherichia coli* investigated the effect of temperature on maturation times by growing cells at 37 and 32 °C, observing large changes in maturation times with temperature.^9^ In this work, we carried out a systematic characterization of FP maturation kinetics for a collection of commonly used FPs in budding yeast, a model eukaryote that grows optimally at 30 °C. To avoid perturbations in cell physiology and growth (which may affect FP maturation kinetics), we developed an experimental approach that does not involve the use of translation inhibitors. Instead, we combined the optogenetic induction of FP expression with mathematical modeling to infer FP maturation rates in a minimally invasive manner.

Our results showed that FP maturation times can vary substantially, even among proteins of the same color. For instance, Cerulean was one of the fastest FPs that we tested, whereas the other two cyan proteins (mTurquoise2 and mTFP1) were among the slowest and were also fitted with a two-step maturation model. Although mTurquoise2 is avGFP derived, previous work^9^ has already indicated that its maturation kinetics cannot be captured by a single exponential. On the other hand, mTFP1 is not an avGFP derivative,^42^ and the maturation process of its chromophore is unknown. Our results suggest that mTFP1 maturation is complex and slow. The estimates of Cerulean and mTurquoise2 maturation half-times agree well with estimates obtained from *E. coli* grown at 32 °C.^9^ However, the *in vitro* characterization of mTFP1 maturation^43^ suggested a much shorter maturation half-time of 15 min, highlighting the discrepancies between *in vitro* and *in vivo* FP studies. Slow cyan FP maturation needs to be taken into account when these proteins are used for the construction of FRET-based biosensors.^6,8^

The green FPs derived from avGFP are among the fastest-maturing FPs known, and therefore, both sfGFP and pH-tdGFP are fast-maturing proteins, with the former being the fastest protein in our collection. An *in vivo* maturation half-time very close to our estimate has been previously obtained in budding yeast.^25^ Interestingly, the maturation of an sfGFP variant very similar to ours was distinctly slower in *E. coli*,^9^ which exemplifies the fact that FP maturation kinetics can vary among organisms. The maturation of pH-tdGFP has not been quantified before, and our results suggest that it is a fast-maturing FP, in agreement with the fact that this is a tandem dimer of two pH-resistant sfGFPs.^44^

Out of the yellow-green FPs that we tested, mNeonGreen and mCitrine showed faster maturation compared to mVenus. Both the mNeonGreen and mVenus maturation half-times are comparable with the *E. coli*-based estimates at 32 °C^9^ (using the nomenclature of our Venus variant, it is Venus JBC in ref (9)). For mCitrine, the *in vivo* maturation half-time had not been determined before. Our estimate is in line with the *in vitro* maturation half-time of the protein at 37 °C (11.5 min^45^). Fast-maturing yellow-green FPs have an advantage over GFP variants, as they can be jointly expressed with cyan FPs when two fast gene expression readouts are needed (*e.g.,* in studies of extrinsic noise^46^).

Red- and far-red-emitting FPs derived from DsRed (mCherry and tdTomato) and TagRFP (mKate2) are known to display complex maturation dynamics through the slow formation of a green/blue fluorescent intermediate.^2,32,36^ mScarlet-I was developed from a synthetic template based on naturally occurring red FPs (RFPs),^47^ and its maturation process is unknown. According to our results, the maturation of mScarlet-I appears to follow similar steps as other RFPs. The complex and slow maturation process of RFPs/far-RFPs, which requires two-step kinetic models to be captured adequately, makes them inappropriate for studying fast-changing gene expression dynamics. Moreover, the practical brightness of these FPs is severely reduced in fast-dividing cells such as bacteria and yeast, due to the existence of a large fraction of immature precursors that are always present in the cell. Still, due to the fact that RFPs or far-RFPs are frequently used as acceptors in FRET-based biosensors, knowledge of their maturation rates is important, as the relative maturation speed of the donor and acceptor is critical for the efficiency of an FRET sensor.^6−8^ Our kinetic models of RFP/far-RFP maturation contain two maturation steps, which lead to nonexponential maturation dynamics and no closed-form expression for the maturation half-time, which needs to be obtained *via* simulation. Moreover, obtaining reliable estimates for the maturation rates of the individual steps is difficult in practice, as our mathematical analysis showed. For this reason, we reported a single maturation rate value for both the maturation steps of FPs with a two-step mechanism. This value was able to accurately reproduce the observed maturation dynamics of each FP.

Our results show that mScarlet-I is the fastest-maturing RFP among those tested, with an effective half-time (31 min) comparable to previous estimates from *E. coli* grown at 32 °C.^9^ mCherry is considerably slower, with an overall *in vivo* maturation half-time (52 min) very close to a previous estimate obtained in budding yeast^25^ (56 min, based on individual half-times of 17 and 30 min). Although mKate2 was reported to have an *in vitro* half-time of less than 20 min at 37 °C,^48^ it is a very slow-maturing far-RFP in budding yeast, and therefore impractical for most applications. Finally, the tdTomato *in vivo* maturation half-time (1.5 h) is also considerably longer than the previously known *in vitro* estimate (1 h at 37 °C^49^).

We believe that the maturation rate estimates presented here will be a useful resource for the budding yeast community and should serve as good starting points for FP maturation estimates in organisms grown at similar or lower temperatures. Thanks to the availability of fast optogenetic gene expression systems for a large range of organisms,^29^ we expect that our experimental approach can be easily adapted to study *in vivo* FP maturation in multicellular organisms as well, where the use of protein synthesis inhibitors is impractical.

## Methods

### Plasmid Construction

*E. coli* Dh5α cells were used for plasmid cloning and propagation. All plasmids were constructed using Gibson assembly.^50^ The details of the plasmids used in this study can be found in Table S1 and the sequences of the primers are available in Table S4.

All polymerase chain reaction (PCR) amplifications in this study were performed using Q5 and Phusion polymerases from New England Biolabs and KOD polymerase from Toyobo. The FPs were subcloned into the pDB60 plasmid downstream of the 5× EL222 transcription factor binding sites, a truncated CYC1 promoter, a Kozak sequence, and upstream of the ADH1 terminator (5×BS-CYC180pr-Kozak-FPs-ADH1t). The pBD146 plasmid was modified to remove the mScarlet-I tag, leaving only the EL222-AQTrip transcription factor construct (ACT1pr-VPEL222_AQTrip-CYC1term). All the plasmids were verified by Sanger sequencing (Eurofins genomics, The Netherlands).

### Yeast Strain Construction

All the presented *S. cerevisiae* strains were derived from BY4741 and YSBN6 (Euroscarf, Germany), both were S288C-derived strains. The strains used in this study are listed in Table S2.

The 5× EL222 transcription factor binding sites and EL222-AQTrip transcription factor constructs were integrated, respectively, into the HIS3 and URA3 loci of BY4741. The former was integrated from PCR-linearized pDB60 and pLV12-21 plasmids and the latter from a PacI-digested pLV1 plasmid. sfGFP- and mRFP1-tagged Hta2 were cloned in the YSBN6 background. Each genetic construct was genomically integrated using the classical lithium acetate transformation,^51^ and all the constructs were verified by Sanger sequencing (Eurofins genomics, Netherlands).

### FP Sequences

All the FP sequences used in this study and their characteristic mutations relative to their predecessors are listed in Table S3. All the DNA sequences of proteins expressed in this study have been yeast codon-optimized.

Three cyan FPs were analyzed, Cerulean, mTurquoise2, and mTFP1. The Cerulean protein used in this study was taken from ref (52) and is similar to the previously described mCerulean ME.^9^ Our protein lacks the A206K substitution of mCerulean ME (which prevents dimerization), and contains the neutral K26R mutation. mTurquoise2 was obtained from ref (53), in which the proteins are truncated at the last 11 amino acids to create mTurquoise2Δ. For the characterization of the protein in this work, we reintroduced these amino acids to generate the full-length version. mTFP1 corresponds to the original version of the protein and was taken from ref (54).

Two green FPs were analyzed, sfGFP and pH-tdGFP. The sfGFP protein characterized in this study is identical to the original with the exception of the previously introduced mutation A206R, which prevents dimerization.^25^ pH-tdGFP is a tandem dimer of two pH-resistant sfGFPs separated by a flexible 22 amino acid linker. These sfGFPs differ from the original by two mutations, N149Y and Q204H.^44^

Three yellow-green FPs were analyzed, mVenus, mCitrine, and mNeonGreen. The mVenus used in this study corresponds to the original mVenus,^55^ also called mVenus JBC in ref (9), and presents the H231L neutral mutation, which was introduced in the EYFP predecessor.^9^ Our mCitrine, taken from ref (27), is equivalent to the original version^56^ with the exception of the F64L substitution, which is known to improve maturation.^57^ mNeonGreen was derived from the tetrameric LanYFP of *Branchiostoma lanceolatum* and the original sequence was used.^58^

Four RFPs were analyzed, mScarlet-I, mCherry, tdTomato, and mKate2. mScarlet-I was taken from ref (59) and is identical to the original.^47^ The mCherry presented in our work was taken from ref (60). tdTomato was also taken from ref (53) with no further mutations. mKate2 was taken from ref (27) without modifications.

### Growth Conditions

For all experiments involving the EL222-AQTrip system, cells were grown in YNB–His medium (Formedium) supplemented with 2% glucose (Sigma-Aldrich). For the estimation of histone dynamics during the cell cycle, cells were grown in minimal medium^61^ supplemented with 2% glucose. Note that the average division time in YNB–His is shorter than in the minimal medium (80 *vs* 100 min). Batch cultivation was carried out at 30 °C with shaking at 300 rpm. Exponentially growing cells were used for all the experiments.

### Microscopy

All microscopy experiments were performed using inverted fluorescence microscopes (Eclipse Ti-E, Nikon Instruments). The temperature was kept constant at 30 °C using a microscope incubator (Life Imaging Services). For all the experiments, a 100× Nikon S Fluor (N.A. = 1.30) objective was used. Images were recorded using the iXon Ultra 897 DU-897-U-CD0-#EX cameras (Andor Technology). Fluorescence measurements were performed using a light-emitting diode (LED)-based excitation system (pE2; CoolLED Limited and Lumencor, AURA). For green fluorescent protein (GFP) measurements, cells were excited at 470 nm (excitation filter: 450–490 nm, dichroic: 495 nm, and emission filter: 500–550 nm). For yellow fluorescent protein (YFP) measurements, cells were excited at 500 nm (excitation filter: 490–510 nm, dichroic: 515 nm, and emission filter: 520–550 nm). For RFP measurements, cells were excited at 565 nm (excitation filter: 540–580 nm, dichroic: 590 nm, and emission filter: 600–650 nm). To activate the EL222-AQTrip system, we used an LED light source centered at 440 nm (pE2; CoolLED Limited) and further filtered at 420–450 nm. During brightfield imaging a long-pass (600 nm) filter was used to prevent unwanted activation of the EL222 system. The Nikon perfect focus system was used to prevent loss of focus.

For all the microscopy experiments involving the EL222 system, batch cultures were grown in the dark. Cell preparation and experiment setups were also conducted in the dark or under a red light in order to prevent unwanted activation of the EL222 system.

For the measurement of fluorescence increase after light stimulation for the estimation of the maturation time, exponentially growing cells were loaded into a microfluidic device^31^ and were continuously fed fresh warm medium at 3.6 μL/min. For each experiment, multiple nonoverlapping *XY* positions were recorded. For each position, unless otherwise stated, the activation of the EL222-AQTrip system and the recording of brightfield and fluorescence images were performed every 5 min. For the EL222 system activation, light pulses of 1 s at ∼100 mW/cm^2^ were used.

For the measurement of fluorescence intensity of cells expressing Hta2-sfGFP or Hta2-mRFP1, exponentially growing cells were placed under a prewarmed agarose pad (minimal medium, 2% glucose, and 1% agarose). Brightfield and fluorescence imaging for multiple *XY* positions were performed every 5 min.

For the estimation of the transcriptional + translational delay (τ_1_ + τ_2_, *cf.*Mathematical Modeling section below) using CHX, exponentially growing cells at OD = 0.1 were incubated in the dark for 30 min at 30 °C in plastic well plates for inverted fluorescence microscopy (Ibidi) treated with concanavalin A (1 mg/mL). The wells were then washed twice with prewarmed medium (+2% glucose) and placed under the microscope. For each well, multiple nonoverlapping *XY* positions were recorded, and for each position, the activation of the EL222 system and the recording of brightfield and fluorescence images were performed every 3 min. A final concentration of 25 μg/mL CHX (diluted in H2O) was then added to the wells at the indicated time.

For the measurement of fluorescence dynamics after CHX addition, cells expressing sfGFP, mVenus, mCherry, and mKate2 under the control of the constitutive TEF1 promoter were incubated in plastic wells as described above, with brightfield and fluorescence images taken every 5 min. A final concentration of 25 μg/mL CHX (diluted in H_2_O) was added to the wells at the indicated time.

### Image Analysis

For each experiment, the fluorescence channel images were background corrected using the rolling ball background subtraction plugin in ImageJ. Cell segmentation and tracking were performed on the brightfield channel using semi-automatic ImageJ plugin BudJ.^62^ The cell volume output of BudJ was used for further analysis. For fluorescence analysis, the background-corrected images were analyzed using a custom-made Python script and the segmentation boundaries were detected using BudJ.

To monitor the single-cell fluorescence dynamics after the activation of EL222-AQTrip, we quantified at each time point the mean cellular fluorescence (mean pixel intensity) of individual mother cells that were present from the beginning to the end of an experiment (Cerulean: *n* = 60, mTurquoise2: *n* = 62, sfGFP: *n* = 55, pHtdGFP: *n* = 57, mTFP1: *n* = 48, mVenus: *n* = 48, mCitrine: *n* = 47, mNeonGreen: *n* = 50, mCherry: *n* = 52, tdTomato: *n* = 39, mKate2: *n* = 53, and mScarlet-I: *n* = 44). For each *XY* position, the segmentation data from BudJ and the corresponding background-corrected images were read in a custom-made Python script. The segmentation information from BudJ was used to generate a mask of the corresponding cell at each time frame. For each cell and time point, the cell mask was applied to the fluorescence channel image and the mean pixel intensity inside the mask of the cell was calculated. Given the very small leakage of the EL222-AQTrip system, cell fluorescence prior to light induction was only due to autofluorescence. To remove the small contribution of autofluorescence in the measurements after light induction, we subtracted from each single-cell fluorescence time series its value at *t* = 0, when no FP is yet present. Assuming that the concentration of the FPs in the mother and in the bud is the same, the corrected mean fluorescence intensity of a mother cell was used as a proxy of the FP concentration in that cell.

For quantification of Hta2-sfGFP and Hta2-mRFP1 fluorescence dynamics during the cell cycle, the same method described above was used to locate and track cells over time, and the sum of pixel intensities inside the mask of the cell was used as a proxy for Hta2-FP abundance. To obtain single-cell Hta2-FP abundance profiles on a common time axis representing normalized cell cycle progression, we first split every cell cycle time series into two parts, one from karyokinesis to the following budding event and the other from the budding event to the next karyokinesis. Budding events were annotated based on the appearance of a dark spot on the mother cell membrane, and karyokinesis was annotated based on the first frame when the nucleus of the mother cell and the nucleus of the bud are completely detached. We then linearly interpolated the first part of every cell cycle time series with a fixed number of equidistant points, and did the same with the second part. The number of points chosen for the interpolation of the first and the second part of each cell cycle time series were calculated based on the ratio of the average durations of the first part and the second part of the cell cycle. For both Hta2-sfGFP data and Hta2-mRFP1 data, we used 30 points to interpolate the first part and 50 points to interpolate the second part of the cell cycle. For subsequent plotting, we then removed the last 10 points of each interpolated time series to exclude the short time interval where the nucleus was already partially in the daughter cell (which was not segmented). We then aligned the interpolated points of each cell cycle on a common (relative) time grid with 70 points between 0 to 1, representing a normalized time axis of cell cycle progression.

### Western Blots

To estimate the total expression delay (τ_1_ + τ_2_), batch cultures were grown in the dark before being subjected to blue illumination using two breadboards each carrying five blue LEDs (Lumex, part no. SSL-LX5093USBC, 470 nm) oriented toward the cultures. Protein extracts were prepared from exponentially growing cells (OD_600_ = 1) which were sampled every 5 min (2.5 OD) and directly treated with CHX at a final concentration of 25 μg/mL (diluted in H_2_O) to stop translation. Proteins were extracted and denatured following the standard alkaline lysis as previously described.^63^

To quantify sfGFP and mKate2 abundance in response to CHX treatment, batch cultures were grown and protein extracts were prepared from exponentially growing cells (OD_600_ = 1) which were sampled every 80 min. The cultures were treated once with CHX (same concentration as mentioned above) 80 min after the first sample. Proteins were extracted after cell fixation with TCA (at a final concentration of 6%). For protein extraction, the fixed cells were incubated on ice for 10 min, washed with cold acetone, and the pellets were air-dried. Subsequently, the cells were lysed by bead beating in urea buffer [50 mM Tris HCl pH 7.5, 5 mM ethylenediaminetetraacetic acid, 6 M urea, and 1% sodium dodecyl sulphate (SDS)], after which samples were shaken (800 rpm) for 10 min at 65 °C and centrifuged for 5 min at 4 °C.

In both experiments, proteins denatured in SDS sample buffer were resolved on 12% SDS-polyacrylamide gel electrophoresis gels and transferred to polyvinylidene fluoride membranes, which were subsequently probed with the following primary antibodies: anti-RFP (Thermo Fisher Scientific, Rabbit polyclonal, #R10367), anti-GFP (Abcam, Rabbit polyclonal, #ab6556), or anti-α-Tubulin (Abcam, Rabbit monoclonal, #ab184970) and secondary antibody: anti-rabbit IgG Superclonal recombinant secondary antibody HRP conjugate (Thermo Fisher, Goat polyclonal, #A27036).

ImageJ software (v.1.52n, Java 1.8.0_202) was used to perform the relative abundance of FPs *Q*_fp_ on the Western blot TIFF images. After converting the images to grayscale, we selected a region of interest corresponding to the largest protein band across the row, and measured the mean gray values of the protein of interest POI and the α-tubulin loading control LC and their respective background mean gray values BC_p_ and BC_l_. The latter were measured below or above each band where no stain was present on the blots. The relative abundance of FPs Q_fp_ is defined as
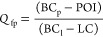


### Mathematical Modeling

The DDEs describing the time evolution of the abundances of the modeled species for the one-step maturation model are
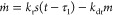
1a
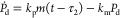
1b
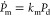
1cwhere *m* denotes the mRNA, *P*_d_ is the dark (immature) precursor, and *P*_m_ is the mature (fluorescent) form of the FP. *s*(*t*) denotes the Heaviside step function (*i.e.,s*(*t*) = 0 for *t* < 0 and *s*(*t*) = 1 for *t* ≥ 0), which models the step-like activation of the EL222-responsive promoter. All states are assumed to be zero for *t* < τ_1_.

The equations corresponding to the two-step maturation model are
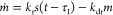
2a
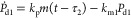
2b
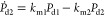
2c
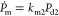
2dwhere *P*_d1_ and *P*_d2_ denote the first and second immature precursors of the fluorescent form *P*_m_, respectively.

The DDEs contain two delay parameters, τ_1_ and τ_2_. The first represents the time required for EL222 promoter activation following the application of light, and the second represents the delay between the appearance of FP mRNA and the production of the immature FP species. τ_1_ was estimated in ref (59) to be around 2 min. To estimate τ_2_, we used Western blotting to locate the moment of appearance of immature protein following light stimulation (Figure S2, Supporting Information Note 2) for three different FPs (fast and slow maturing), and cross-validated the results with CHX addition at different time points after light induction for two FPs (Figure S3, Supporting Information Note 2). Both approaches allowed us to conclude that the total delay between EL222 activation and the appearance of immature protein (τ_1_ + τ_2_) is very close to 6 min, irrespective of the tested FP. From this total delay, we estimated τ_2_ to be around 4 min. Overall, the precise estimation of τ_1_ + τ_2_ is more critical for fast-maturing FPs than for slow ones (Supporting Information Note 7).

Due to the fact that our experimental data consists of concentration measurements in mother cells present from the beginning to the end of an experiment, we complemented the abundance model (eqs 1a–1c, 2a–2d) with a single-cell volume model that describes growth and division processes for a mother cell over consecutive cell cycles. A cell cycle starts at the moment a bud divides and ends at the next bud division. We assume that during the *n*-th cell cycle (*c*_*n*_) of duration *T*_*c*_*n*_,_ a cell grows linearly from an initial volume *V*_0,*c*_*n*__ to a volume *V*_1,*c*_*n*__ with a growth rate μ_*c*_*n*__ (Figure 1E)
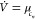
3

Even though the instantaneous growth rate of individual yeast cells is known to fluctuate during the cell cycle,^62,64^ assuming a constant growth rate for our model is sufficient because growth rate fluctuations are averaged out in an asynchronously growing cell population. At the end of the cell cycle, the cell loses a volume *V*_d,*c*_*n*__ (Figure 1E) due to the detachment of the newborn daughter cell. We can then define the fraction *f*_*c*_ of the mother–bud volume that remains after division
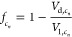
4

Assuming that the mRNA and FP protein species are uniformly distributed in the mother–bud volume, the abundance of mRNA, *P*_d_, and *P*_m_ at the start of the following cell cycle is reset by the fraction *f*_*c*_
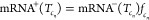
5a
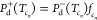
5b
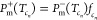
5c
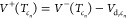
5dwhere the minus and plus superscripts denote the value of the corresponding variable, infinitesimally before and after division. The following cell cycle (*c*_*n*+1_) then starts with an initial volume *V*_0,*cn*+1_ = *V*^+^(*T*_*c*_*n*__), and a new set of parameters *T*_*c*_*n*+1__, μ_*c*_*n*+1__ and *V*_*d*,*c*_*n*+1__. For each cell cycle, these three parameters are sampled from a multivariate log-normal distribution. This distribution was fitted to experimentally determined values of *T*, μ, and *V*_d_, obtained from the same mother cells whose fluorescence was tracked over time.

Given that asymmetric division leads to mother cell aging, which is accompanied by a gradual increase in cell size over consecutive divisions,^65^ the following condition was applied when sampling a set of parameters, to ensure that the volume lost at the end of a cell cycle of our simulated cells is less than the volume gained during that cell cycle, that is*,* a cell grows in volume over successive divisions (Figure S4E,F)
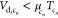
6

This condition is already fulfilled in most cases when sampling from the estimated multivariate distribution of cell cycle parameters, but the explicit enforcement of the condition ensures that every simulated cell satisfies it.

To account for the population asynchronicity, each simulated cell starts at a random point in its first cell cycle with an initial volume
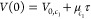
7where τ is uniformly distributed in [0,*T*_*c*_1__]. For each simulated cell, the initial starting volume of the first cell cycle (*V*_0,*c*_1__) is sampled from a normal distribution fitted to the experimentally measured mother cell volumes at *t* = 0 (start of EL222-AQTrip activation).

Combining the abundance model with the volume model, we could then calculate the FP concentration *P*_m,*C*_ of the mature FP for each simulated cell over a time horizon corresponding to the observation horizon in our experiments
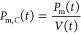
8

Because the volume dynamics introduces stochasticity in our model, 500 cells were simulated in order to produce a population-averaged FP concentration profile that was fitted to the experimental data.

### Estimation of Parameter Distributions for the Volume Model

To generate the probability distributions from which the cell cycle parameters of the volume model (*T*_*c*_, μ_*c*_ and *V*_d,*c*_) were drawn, we first determined the empirical distributions of these quantities for the same cells whose fluorescence was quantified (number of cell cycles analyzed: Cerulean: *n* = 100, mTurquoise2: *N* = 91, sfGFP: *n* = 101, pHtdGFP: *n* = 69, mTFP1: *n* = 91, mVenus: *n* = 99, mCitrine: *n* = 87, mNeonGreen: *n* = 102, mCherry: *n* = 89, tdTomato: *n* = 63, mKate2: *n* = 71, and mScarlet-I: *n* = 103). *T*_*c*_ was defined as the time between two divisions (cytokinesis events), μ_*c*_ as the average growth rate between two divisions, and *V*_d,*c*_ was the volume of the bud at division. Cytokinesis events were identified by the darkening of the bud neck and the slight movement of the bud away from the mother cell. The average growth rate μ_*c*_ was calculated by subtracting the volume of a mother cell after a cytokinesis (*V*_0_) from the mother + bud volume at the next cytokinesis (*V*_1_) and dividing by *T*_*c*_. Considering the right skew of these empirical distributions (Figure S4B–D), we fitted a multivariate log-normal distribution to *T*_*c*_, μ_*c*_ and *V*_d,*c*_ by calculating the empirical means and covariance matrix of the logarithms of *T*_*c*_, μ_*c*_ and *V*_d,*c*_.

### Maximum Likelihood Parameter Estimation

Following the light induction of FP expression, the fluorescence measurements corresponding to the FP concentration in mother cells were obtained every 5 min up to 200 min post-induction. We denote the set of fluorescence measurements by {*y*(*t*_*n*_), *n* = 1,...,*N*} and the model-based prediction of population-averaged FP concentration over 500 simulated cells using the parameter vector θ by *P̅*_m,*c*_(*t*,θ), *t* ≥ 0. We further assume that our observations are corrupted by additive independent, normally distributed noise samples, leading to an observation model of the form *ŷ*(*t*_*n*_,θ) = *P̅*_m,*c*_(*t*_*n*_,θ) + ε_*n*_, with ε_*n*_ ∼ *N*(0,σ^2^), where σ = 0.02·max({*y*(*t*_*n*_), *n* = 1,...,*N*}). The value of σ was chosen empirically to correspond to the level of measurement variability observed due to the finite number of cells tracked in each experiment and day-to-day variability in experimental measurements. Under this noise model, the maximization of the likelihood with respect to θ is equivalent to the minimization of the sum of squares objective function
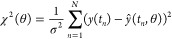
9

### Profile Likelihood Analysis

To verify the practical identifiability of the maturation rates and derive confidence intervals, we made use of the profile likelihood methodology.^66^ Given the definition of χ^2^(θ) in ref (9) and assuming that we are interested in θ_*i*_, the *i*-th component of the vector θ, the (negative) profile log-likelihood is defined as
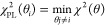
10

Practical identifiability of θ_*i*_ can be investigated by examining the (1 – *a*)% confidence interval for θ_*i*_ for a given significance level *a*: if the confidence interval is finite at the desired confidence level, the parameter θ_*i*_ is deemed practically identifiable at the given confidence level.^66^ Here, we considered *a* = 0.05 and constructed 95% confidence intervals for the maturation rates in our FP models. These confidence intervals are given by the set of θ_*i*_ values for which χ_PL_^2^(θ_*i*_) is smaller than χ^2^(θ*) + Δ_1;1−α_, where θ* is the parameter vector that minimizes χ^2^(θ) and Δ_1;1−α_ is the (1 – *a*)% quantile of the χ^2^ distribution with 1 degree of freedom. Symbolically
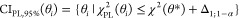
11

The calculation of χ_PL_^2^(θ_*i*_) over a grid of θ_*i*_ values requires repeated minimization of the function χ^2^(θ). In turn, each optimization run requires multistarting to ensure that the global minimum will be reached. Given that the abundance model contained a small number of unknown parameters, we avoided running optimization by estimating χ^2^(θ) on a dense grid in the space of locally identifiable model parameters (*i.e.*, setting *k*_r_ = *k*_p_, *cf.*Supporting Information Note 5) with 60 points per dimension, and evaluating the profile likelihoods and confidence intervals with the grid-based values of χ^2^(θ). For one-step maturation models, θ = [*k*_dr_*k*_p_*k*_m_]. For two-step maturation models, θ = [*k*_dr_*k*_p_*k*_m1_], while *k*_m2_ was set equal to *k*_m1_ (*cf.*Supporting Information Note 5). The search space for *k*_m_ (and, correspondingly, *k*_m1_) was bounded from above at 0.1386 (corresponding to a maturation half-time of 5 min), with no lower bound, while the search space for *k*_dr_ was limited between 0.0462 and 0.1386, corresponding to mRNA degradation half-lives between 5 and 15 min, in line with the available experimental evidence on the GFP mRNA degradation half-life in budding yeast.^67^

## Supporting Information Available

Lists of FPs, plasmids, strains and primers used; details on light induction tests, experimental delay characterization, determination of cell cycle parameters and model fits; and description of light induction protocol, estimation of time delays, modeling of histone synthesis, model selection, simplified concentration model, and sensitivity analysis (PDF)
Summaries of the cell cycle and fluorescence data from microscopy experiments for each tested FP (ZIP)
