# Supplementary material for: Systematic In Vivo Characterization of Fluorescent Protein Maturation in Budding Yeast
Source: ACS Synth Biol. 2022 Feb 18;11(3):1129–41. doi: 10.1021/acssynbio.1c00387 (PMC8938947; doi:10.1021/acssynbio.1c00387)
Supplement: Supplementary file 1 — sb1c00387_si_001.pdf [file sb1c00387_si_001.pdf]

Supporting Information for

Systematic *in vivo* characterization of fluorescent protein maturation in budding yeast

Paolo Guerra[1], Luc-Alban Vuillemenot[1], Brady Rae[1], Valeriia Ladyhina[1],

Andreas Milias-Argeitis[1,*]

[1] Molecular Systems Biology, Groningen Biomolecular Sciences & Biotechnology Institute, University of Groningen, 9747 AG Groningen, Netherlands

*Correspondence: a.milias.argeitis@rug.nl

Supplementary Tables

Supplementary Table 1.

List of plasmids used in this study.

Plasmid	Backbone	Insert	Source
pDB60	pFA6- *his3MX6*	5xBS-*CYC180*pr-Kozak-mKate2-*ADH1*t	(1)
pDB146	pKERG106	ACT1pr-mScarletI-VPEL222_AQTrip-CYC1t	(2)
pLV1	pDB146	ACT1pr-VPEL222_AQTrip-CYC1t	This study
pLV12	pDB60	5xBS-*CYC180*pr-Kozak-pH-tdGFP-*ADH1*t	This study
pLV13	pDB60	5xBS-*CYC180*pr-Kozak-sfGFP-*ADH1*t	This study
pLV14	pDB60	5xBS-*CYC180*pr-Kozak-mCherry-*ADH1*t	This study
pLV15	pDB60	5xBS-*CYC180*pr-Kozak-mTFP1-*ADH1*t	This study
pLV16	pDB60	5xBS-*CYC180*pr-Kozak-mVenus- *ADH1*t	This study
pLV17	pDB60	5xBS-*CYC180*pr-Kozak-mNeongreen-*ADH1*t	This study
pLV18	pDB60	5xBS-*CYC180*pr-Kozak-Cerulean- *ADH1*t	This study
pLV19	pDB60	5xBS-*CYC180*pr-Kozak-mTurquoise2-*ADH1*t	This study
pLV20	pDB60	5xBS-*CYC180*pr-Kozak-tdTomato-*ADH1*t	This study
pLV21	pDB60	5xBS-*CYC180*pr-Kozak-mScarletI-*ADH1*t	This study

Supplementary Table 2.

List of yeast strains used in this study.

Strain	Genotype	Source
BY4741	MATa *his3Δ1 leu2Δ0 met15Δ0 ura3Δ0*	Euroscarf
YSBN6	MATa FY3 HO::HphMX4	Euroscarf
DBY73	BY4741, *his3Δ*::5xBS-*CYC180*pr-Kozak-mKate2-*ADH1*t-*HIS3MX* (pDB60)	(1)
DBY104	DBY42, *his3Δ*::5xBS-*CYC180*pr-Kozak-mCitrine-*ADH1*t-*HIS3MX* (pDB110)	(1)
YLV2	BY4741, *URA3::*ACT1pr-VPEL222_AQTrip-CYC1t (pLV1)	This study
YLV3	YLV2, *his3Δ*::5xBS-*CYC180*pr-Kozak-mKate2-*ADH1*t-*HIS3MX* (pDB60)	This study
YLV4	YLV2, *his3Δ*::5xBS-*CYC180*pr-Kozak-pH-tdGFP-*ADH1*t-*HIS3MX* (pLV12)	This study
YLV5	YLV2, *his3Δ*::5xBS-*CYC180*pr-Kozak-sfGFP-*ADH1*t-*HIS3MX* (pLV13)	This study
YLV6	YLV2, *his3Δ*::5xBS-*CYC180*pr-Kozak-mTFP1-*ADH1*t-*HIS3MX* (pDB60)	This study
YLV7	YLV2, *his3Δ*::5xBS-*CYC180*pr-Kozak-mVenus-*ADH1*t-*HIS3MX* (pLV15)	This study
YLV8	YLV2, *his3Δ*::5xBS-*CYC180*pr-Kozak-mCherry-*ADH1*t-*HIS3MX* (pLV14)	This study
YLV9	YLV2, *his3Δ*::5xBS-*CYC180*pr-Kozak-mCitrine-*ADH1*t-*HIS3MX* (DBY104)	This study
YLV29	YLV2, *his3Δ*::5xBS-*CYC180*pr-Kozak-mNeongreen-*ADH1*t-*HIS3MX* (pLV17)	This study
YLV30	YLV2, *his3Δ*::5xBS-*CYC180*pr-Kozak-tdTomato-*ADH1*t-*HIS3MX* (pLV20)	This study
YLV31	YLV2, *his3Δ*::5xBS-*CYC180*pr-Kozak-mTurquoise2-*ADH1*t-*HIS3MX* (pLV19)	This study
YLV32	YLV2, *his3Δ*::5xBS-*CYC180*pr-Kozak-Cerulean-*ADH1*t-*HIS3MX* (pDB60)	This study
YLV33	YLV2, *his3Δ*::5xBS-*CYC180*pr-Kozak-mScarletI-*ADH1*t-*HIS3MX* (pLV18)	This study
YLV34	YSBN6, Hta2::mRFP1-Ble	This study
YLV35	YSBN6, Whi5::mCherry-Ble Hta2::sfGFP-KanMX	This study
YLV36	YSBN6, HO::pTEF1-mKate2-KanMx	This study
YLV37	YSBN6, HO::pTEF1-sfGFP-KanMX Whi5::mCherry-Ble	This study

Supplementary Table 3.

List of fluorescent proteins sequences and modifications relative to their FP ancestor (numbering relative to the ancestor's sequence).

**Fluorescent protein**	**Sequence**	**Mutations relative to the corresponding FP ancestor**	**Ref.**
Cerulean	MVSKGEELFTGVVPILVELDGDVNGHRFS VSGEGEGDATYGKLTLKFICTTGKLPVPW PTLVTTLTWGVQCFARYPDHMKQHDFFK SAMPEGYVQERTIFFKDDGNYKTRAEVKF EGDTLVNRIELKGIDFKEDGNILGHKLEYN AISDNVYITADKQKNGIKAHFKIRHNIEDGS VQLADHYQQNTPIGDGPVLLPDNHYLSTQ SALSKDPNEKRDHMVLLEFVTAAGITLGM DELYK*	M1_S2insV, K26R, F64L, S65T, Y66W, S72A, Y145A, N146I, H148D, M153T, V163A, N164H & H231L<br>(VS. avGFP)	(3)
mTurquoise2	MVSKGEELFTGVVPILVELDGDVNGHKFS VSGEGEGDATYGKLTLKFICTTGKLPVPW PTLVTTLSWGVQCFARYPDHMKQHDFFK SAMPEGYVQERTIFFKDDGNYKTRAEVKF EGDTLVNRIELKGIDFKEDGNILGHKLEYN YFSDNVYITADKQKNGIKANFKIRHNIEDG GVQLADHYQQNTPIGDGPVLLPDNHYLST QSKLSKDPNEKRDHMVLLEFVTAAGITLG MDELYK*	M1_S2insV, F64L, Y66W, S72A, N146F, H148D, M153T, V163A, S175G, A206K & H231L<br>(VS. avGFP)	(4)
mTFP1	MVSKGEETTMGVIKPDMKIKLKMEGNVNG HAFVIEGEGEGKPYDGTNTINLEVKEGAPL PFSYDILTTAFAYGNRAFTKYPDDIPNYFK QSFPEGYSWERTMTFEDKGIVKVKSDISM EEDSFIYEIHLKGENFPPNGPVMQKKTTG WDASTERMYVRDGVLKGDVKHKLLLEGG GHHRVDFKTIYRAKKAVKLPDYHFVDHRIE ILNHDKDYNKVTVYESAVARNSTDGMDEL YK*	K2_L29delinsVSKG, K32_L40del, H80N, L82I, S100T, N101T, Q104A, L110F, A118P, D119N, R161H, F162L, D163K, M165E, L179T, K180G, E182D, P183A, I187R, V196K, I199V, S200K, S202K, Y211H, C213V, S217T, K220R, V224A, L251V, N254S, Y259N, L261T, L261_L262insDG, L262M, P263D, S264E, S264_Q265insL, Q265Y & A266K<br>(VS. cFP484)	(5)

sfGFP	MSKGEELFTGVVPILVELDGDVNGHKFSVRGEGEGDATNGKLTLKFICTTGKLPVPWPTLVTTLTYGVQCFSRYPDHMKRHDFFKSAMPEGYVQERTISFKDDGTYKTRAEVKFEGDTLVNRIELKGIDFKEDGNILGHKLEYNFNSHNVYITADKQKNGIKANFKIRHNVEDGSVQLADHYQQNTPIGDGPVLLPDNHYLSTQSRLSKDPNEKRDHMVLLEFVTAAGITHGMDELYK*	S30R, Y39N, F64L, S65T, Q80R, F99S, N105T, Y145F, M153T, V163A, I171V & A206R<br>(VS. avGFP)	(6)
pH-tdGFP	MSKGEELFTGVVPILVELDGDVNGHKFSVRGEGEGDATNGKLTLKFICTTGKLPVPWPTLVTTLTYGVQCFSRYPDHMKRHDFFKSAMPEGYVQERTISFKDDGTYKTRAEVKFEGDTLVNRIELKGIDFKEDGNILGHKLEYNFNSHYVYITADKQKNGIKANFKIRHNVEDGSVQLADHYQQNTPIGDGPVLLPDNHYLSTHSVLSKDPNEKRDHMVLLEFVTAAGITHGHGTGSTGSGSSGTASSEDNNMALFTGVVPILVELDGDVNGHKFSVRGEGEGDATNGKLTLKFICTTGKLPVPWPTLVTTLTYGVQCFSRYPDHMKRHDFFKSAMPEGYVQERTISFKDDGTYKTRAEVKFEGDTLVNRIELKGIDFKEDGNILGHKLEYNFNSHYVYITADKQKNGIKANFKIRHNVEDGSVQLADHYQQNTPIGDGPVLLPDNHYLSTHSVLSKDPNEKRDHMVLLEFVTAAGITHGMDELYK*	S30R, A37_T38insTNGKLTLKFICTTGKLPVPWPTLVTTL, Y39_G40insGVQCFSRYPDHMKRHDFFKSAMPEGYVQERTISFKDDGTYKTRAEVKFEGDTLVNRIELKGIDFKEDGNILGHKLEYNFNSHYVYITADKQKNGIKANFKIRHNVEDGSVQLADHYQQNTPIGDGPVLLPDNHYLSTHSVLSKDPNEKRDHMVLLEFVTAAGITHGHGTGSTGSGSSGTASSEDNNMALFTGVVPILVELDGDVNGHKFSVRGEGEGDATN, F64L, S65T, Q80R, F99S, N105T, Y145F, N149Y, M153T, V163A, I171V, Q204H & A206V<br>(VS. avGFP)	(7)
mVenus	MVSKGEELFTGVVPILVELDGDVNGHKFSVSGEGEGDATYGKLTLKLICTTGKLPVPWPTLVTTLGYGLQCFARYPDHMKQHDFFKSAMPEGYVQERTIFFKDDGNYKTRAEVKFEGDTLVNRIELKGIDFKEDGNILGHKLEYNYNSHNVYITADKQKNGIKANFKIRHNIEDGGVQLADHYQQNTPIGDGPVLLPDNHYLSYQSKLSKDPNEKRDHMVLLEFVTAAGITLGMDELYK*	M1_S2insV, F46L, F64L. S65G, V68L, S72A, M153T, V163A, S175G, T203Y, A206K & H231L<br>(VS. avGFP)	(8)
mCitrine	MKGEELFTGVVPILVELDGDVNGHKFSVSGEGEGDATYGKLTLKFICTTGKLPVPWPTLVTTLGYGLMCFARYPDHMKQHDFFKSAMPEGYVQERTIFFKDDGNYKTRAEVKFEGDTLVNRIELKGIDFKEDGNILGHKLEYNYNSHNVYIMADKQKNGIKVNFKIRHNIEDGSVQLADHYQQNTPIGDGPVLLPDNHYLSYQSKLSKDPNEKRDHMVLLEFVTAAGITHGMDELYK*	V2_S3del, F64L, S65G, V68L, Q69M, S72A, T203Y, A206K & H231L<br>(VS. avGFP)	(1)

mNeongreen	MVSKGEEDNMASLPATHELHIFGSINGVD FDMVGQGTGNPNDGYEELNLKSTKGDLQ FSPWILVPHIGYGFHQYLPYPDGMSPFQA AMVDGSGYQVHRTMQFEDGASLTVNYRY TYEGSHIKGEAQVKGTGFPADGPVMTNSL TAADWCRSKKTYPNDKTIISTFKWSYTTG NGKRYRSTARTTYTFAKPMAANYLKNQP MYVFRKTELKHSKTELNFKEWQKAFTDV MGMDELYK*	M1_S2insVSKGEEDNMA, F15I, R25Q, A45D, Q56H, F67Y, K79V, S100V, F115A, I118K, V140R, T141S, M143K, L144T, D156K, T158S, S163N, Q168R, V171A, N174T, I185Y, F192Y & 220GextMDELYK<br>(VS. LanYFP)	(9)
mScarlet-I	MVSKGEAVIKEFMRFKVHMEGSMNGHEF EIEGEGEGRPYEGTQTAKLKVTKGGPLPF SWDILSPQFMYGSRAFIKHPADIPDYYKQ SFPEGFKWERVMNFEDGGAVTVTQDTSL EDGTLIYKVKLRGTNFPPDGPVMQKKTMG WEASTERLYPEDGVLKGDIKMALRLKDGG RYLADFKTTYKAKKPVQMPGAYNVDRKL DITSHNEDYTVVEQYERSEGRHSTGGMD ELYK*	N6A, R17H, T21S, V22M, H41T, N42Q, V44A, A57S, Q66M, K70R, V71A, Y72F, V73I, K83Y, L85Q, V104A, S111T, Q114E, C117T, F118L, F124L, I125R, V127T, S131P, R153E, E160D, H162K, K163M, K166R, H172R, V175A, E176D, S179T, I180T, M182K, L189M, Y192A, Y194N, S197R, I210V, T217S, H222S, L223T, F224G<br>(VS. DsRed)	(2)
mCherry	MS^MVSKGEENNMAIIKEFMRFKVHMEGS VNGHEFEIEGEGEGRPYEGTQTAKLKVTK GGPLPFAWDILSPQFMYGSKAYVKHPADI PDYLKLSFPEGFKWERVMNFEDGGVVTV TQDSSLQDGEFIYKVKLRGTNFPSDGPVM QKKTMGWEASSERMYPEDGALKGEIKQR LKLKDGGHYDAEVKTTYKAKKPVQLPGAY NVNIKLDITSHNEDYTIVEQYERAEGRHST GGMDELYK*	MS^ (additional amino acids), R2_S3delinsV, K5_N6insGEEN, N6_V7insMA, V7I, R17H, T21S, H41T, N42Q, V44A, Q66M, V71A, K83L, C117E, F124L, I125R, V127T, T147S, L150M, R153E, V156A, H162K, K163Q, A164R, L174D, V175A, F177V, S179T, I180T, M182K, Y192A, Y194N, D196N, S197I, T217A, H221_H222insSTGGMD, H222E, F224Y & L225K<br>(VS. DsRed)	(10)

tdTomato	MVSKGEEVIKEFMRFKVRMEGSMNGHEF<br>EIEGEGEGRPYEGTQTAKLKVTKGGPLPF<br>AWDILSPQFMYGSKAYVKHPADIPDYKKL<br>SFPEGFKWERVMNFEDGGLVTVTQDSSL<br>QDGTLIYKVKMRGTNFPPDGPVMQKKTM<br>GWEASTERLYPRDGVLKGEIHQALKLKDG<br>GHYLVEFKTIYMAKKPVQLPGYYYVDTKL<br>DITSHNEDYTIVEQYERSEGRHHLFLGHG<br>TGSTGSGSSGTASSEDNNMAVIKEFMRF<br>KVRMEGSMNGHEFEIEGEGEGRPYEGTQ<br>TAKLKVTKGGPLPFAWDILSPQFMYGSKA<br>YVKHPADIPDYKKLSFPEGFKWERVMNFE<br>DGGLVTVTQDSSLQDGTLIYKVKMRGTNF<br>PPDGPVMQKKTMGWEASTERLYPRDGVL<br>KGEIHQALKLKDGGHYLVEFKTIYMAKKPV<br>QLPGYYYVDTKLDITSHNEDYTIVEQYERS<br>EGRHHLFLYGMDELYK*	R2V, S3_S4insK, S4G, K5E,<br>N6E, T21S, V22M,<br>G40_H41insTQTAKLKVTKGGPL<br>PFAWDILSPQFMYGSKAYVKHP<br>ADIPDYKKLSFPEGFKWERVMN<br>FEDGGLVTVTQDSSLQDGTLIY<br>KVKMRGTNFPPDGPVMQKKTM<br>GWEASTERLYPRDGVLKGEIHQ<br>ALKLKDGGHYLVEFKTIYMAKK<br>PVQLPGYYYVDTKLDITS,<br>N42_T43insEDYTIVEQYERSEG<br>RHHLFLGHGTGSTGSGSSGTA<br>SSEDNNMAVIKEFMRFKVRME<br>GSMNGHEFEIEGEGEGRPYEG<br>TQ, V44A, Q66M, V71A, V104L,<br>C117T, F118L, F124M, I125R,<br>V127T, S131P, K163Q, S179T,<br>S197T, T217S,<br>226YextGMDELYK<br><br>(VS. DsRed)	(4)
mKate2	MRSVSELIKENMHMKLYMEGTVNNHHFK<br>CTSEGEGKPYEGTQTMRIKAVEGGPLPFA<br>FDILATSFMYGSKTFINHTQGIPDFFKQSF<br>PEGFTWERVTTYEDGGVLTATQDTSLQD<br>GCLIYNVKIRGVNFPSNGPVMQKKTLGWE<br>ASTETLYPADGGLEGRADMALKLVGGGH<br>LICNLKTTYRSKKPAKNLKMPGVYYVDRR<br>LERIKEADKETYVEQHEVAVARYCDLPSK<br>LGHRGS*	M1_S2insRSV, R32G, K42R,<br>V45A, L79F, I93V, N112D, I115L,<br>N122R, S131P, N143S, M146T,<br>R155E, H157R, S158A, Q159D,<br>Y169H, H171I, S173N, F174L,<br>F192V, H193Y, F194Y, H197R,<br>M216V & K220R, R231_insGS<br><br>(VS. eqFP578)	(1)

Supplementary Table 4.

List of primers used in this study.

Use	Primer name	Primer sequence
Plasmid cloning	sfGFP_fwd	CAAATACATTAATTAAAACAAAATGTCCAAGGGTGAAGAG
	sfGFP_rev	TTATTTAGAAGTGGCGCGCCTTACTTATAAAGCTCGTCCATTC
	mCherry_fwd	CAAATACATTAATTAAAACAAAATGTCTATGGTTAGTAAAGG
	mCherry_rev	TTATTTAGAAGTGGCGCGCCTTATTTGTATAGTTCATCCATGC
	mVenus_fwd	CAAATACATTAATTAAAACAAAATGGTTAGTAAAGGTGAAG
	mVenus_rev	TTATTTAGAAGTGGCGCGCCTTACTTGTATAATTCATCCATACC
	mTFP1_fwd	CAAATACATTAATTAAAACAAAATGGTTTCCAAGGGTGAAG
	mTFP1_rev	TTATTTAGAAGTGGCGCGCCTTACTTGTACAACTCATCCATAC
	pH-tdGFP_fwd	CAAATACATTAATTAAAACAAAATGTCCAAGGGTGAAGAATTATTC
	pH-tdGFP_rev	TTATTTAGAAGTGGCGCGCCTTACTTGTATAATTCATCCATACCG
	mNeongreen_fwd	CAAATACATTAATTAAAACAAAATGGTGAGCAAGGGC
	mNeongreen_rev	TTATTTAGAAGTGGCGCGCCTTACTTGTACAGCTCGTCCAT
	tdTomato_fwd	CAAATACATTAATTAAAACAAATGGTGAGCAAGGGCGAG
	tdTomato_rev	TTATTTAGAAGTGGCGCGCCTTACTTGTACAGCTCGTCCATGC
	mTurquoise2_fwd	CAAATACATTAATTAAAACAAAATGGTGAGCAAGGGCGAGGAGCTG
	mTurquoise2_rev	TTATTTAGAAGTGGCGCGCCTTACTTGTACAGCTCGTCCATGCCGAGAGTGATCCCGGCGGCGGTCAC
	CFP_fwd	CAAATACATTAATTAAAACAAAATGGTGTCTAAAGGTGAAG
	CFP_rev	TTATTTAGAAGTGGCGCGCCTTACTTGTACAACTCATCCATACCTAA
	mScarlet-I_fwd	CAAATACATTAATTAAAACAAAATGGTGAGCAAGGGCGAG
	mScarlet-I_rev	TTATTTAGAAGTGGCGCGCCTTATTTGTACAGCTCGTCCATGC
	pDB110-mCitrine_int_fwd	TCTTGGCCTCCTCTAGTACACTC
	pDB110-mCitrine_int_rev	TATGGCAACCGCAAGAGCC
	pDB60_backbone_fwd	GGCGCGCCACTTCTAAATAAG
	pDB60_backbone_rev	TTTGTTTTAATTAATGTATTTGTGTTTGTGTG

	NLS_VP_EL222AQ_fwd	AACAACTAGTATGGGCCCTAAAAAGAAGCGTAAAG
	Ura3_rev	ACACCGCATATGCTTTACAGTCCT
	Act1_fwd	CTGTAAAGCATATGCGGTGTGAAATAC
	Act1_rev	TAGGGCCCATACTAGTTGTTAATTCAGTAAATTTTCGA
Genomic integration	pDB129 (HIS3_insertion_fwd)	TCTTGGCCTCCTCTAGTACACTCTATATTTTTTTATGCCTCGGTAATGAGAAACCATTATTATCATGACATTAACC
	pDB130 (HIS3_insertion_fwd)	TATGGCAACCGCAAGAGCCTTGAACGCACTCTCTACGGATCGATGAATTCGAGCTCG
Sequencing	HIS3_genome_check_fwd	ATGCCTCGGTAATGA
	HIS3_genome_check_rev	ACTCTCACTACGG
	URA3_ter_int_check_fwd	GAACAATAAGACAGGACTGTAAAG
	URA3_ter_int_check_rev	TACATGCATTTACTTATAATACAGTTTT
	EL222_seq_fwd	AGTCCTTTCCCGCAATTTTCTTTTTCT
	pDB60_seq_fwd	GACGACACATGATCATATGGC
	pDB60_seq_rev	TGGTCAATAAGAGCGACCTC
	Seq1_pDB-FPs_fwd	ACATTAGGACCTTTGCAGC
	EL222-mTFP1_seq_fwd	CAGACGATATCCCAAACTACTTCAAG
	EL222-sfGFP_seq_fwd	TAAAAGTGCTATGCCAGAAGGC
	EL222-pH-tdGFP-seq2_fwd	CACAATGTCGAAGATGGTTCAGTTC
	EL222-mCherry_seq_fwd	CTCAATTCATGTATGGGTCAAAGG
	EL222_mVenus_seq_fwd	ATGACTTTTTTAAGTCTGCAATGCC
	EL222-sfGFP_seq_fwd	TAAAAGTGCTATGCCAGAAGGC
	EL222_tdTomato_seq_rev	TTATTTAGAAGTGGCGCGCC
	SeqEL1_fwd	GCCCCCCCGACCGATGTCAG
	SeqEL2_fwd	CTTAGACGGCGAGGACGTGGCG
	Seq1_Ura_int_fwd	ATGTGCTGCAAGGCGATTAAG

Supplementary figures

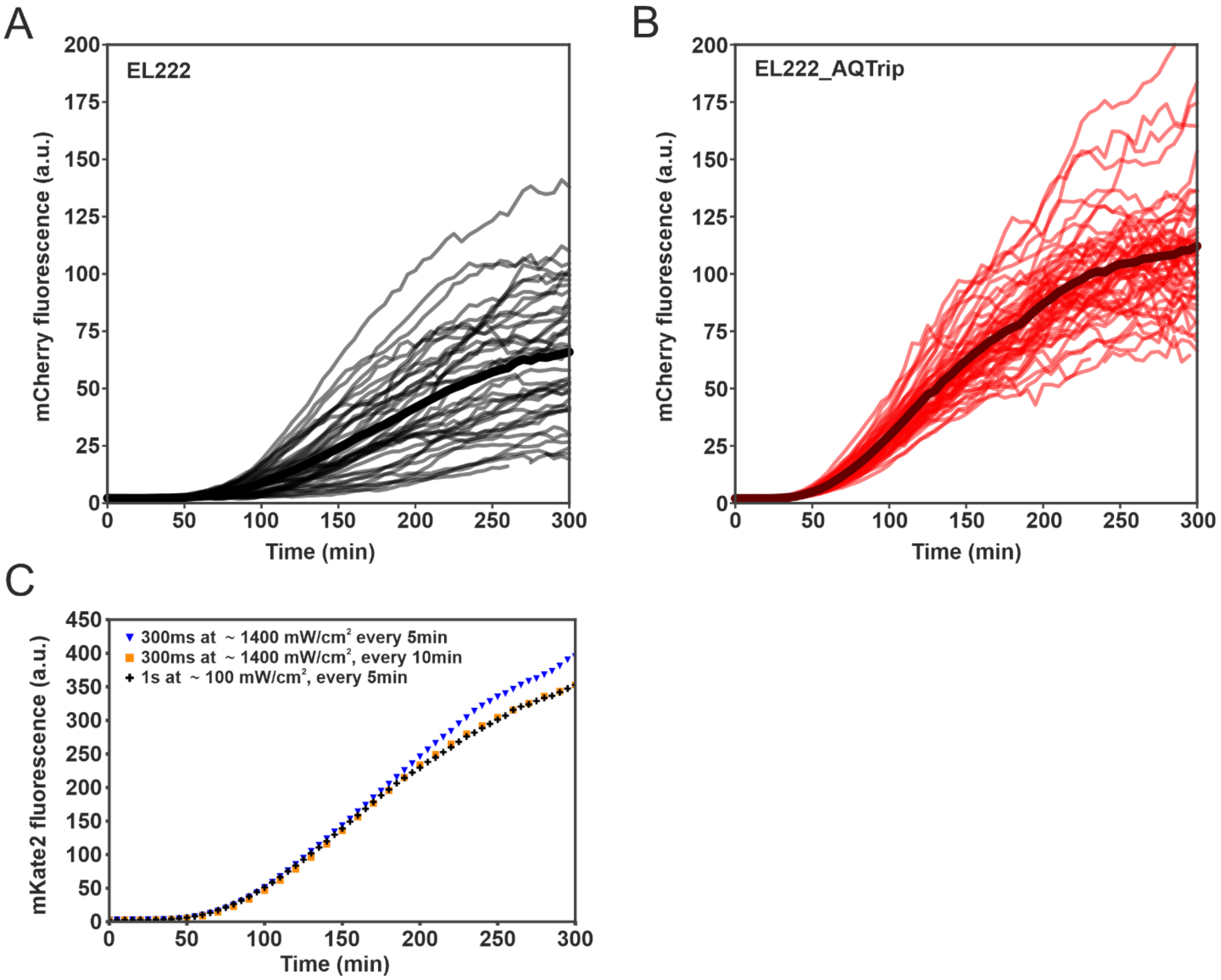

Figure S1

A) Fluorescence intensity dynamics in individual mother cells expressing mCherry driven by the original (wild-type) EL222 system (1) (n=40). Thick black line represents the population mean. The EL222 system was activated at time $t = 0$ by short light pulses (440nm, 300ms at 1400mW/cm^2) every 5 min. **B)** Fluorescence increase in individual mother cells expressing mCherry driven by the EL222_AQTrip system (11) (n = 52). Thick red line represents the population mean. The EL222_AQTrip system was activated at time $t = 0$ by short light pulses (440nm, 300ms at 1400mW/cm^2) every 5 min. Besides achieving higher expression levels than the wild-type system in response to pulsatile excitation thanks to the slow dark reversion of AQTrip, this optogenetic system produces less cell-to-cell variability in induction dynamics. **C)** Comparison of average fluorescence dynamics in cells containing mKate2 driven by the EL222_AQTrip system with different light activation settings.

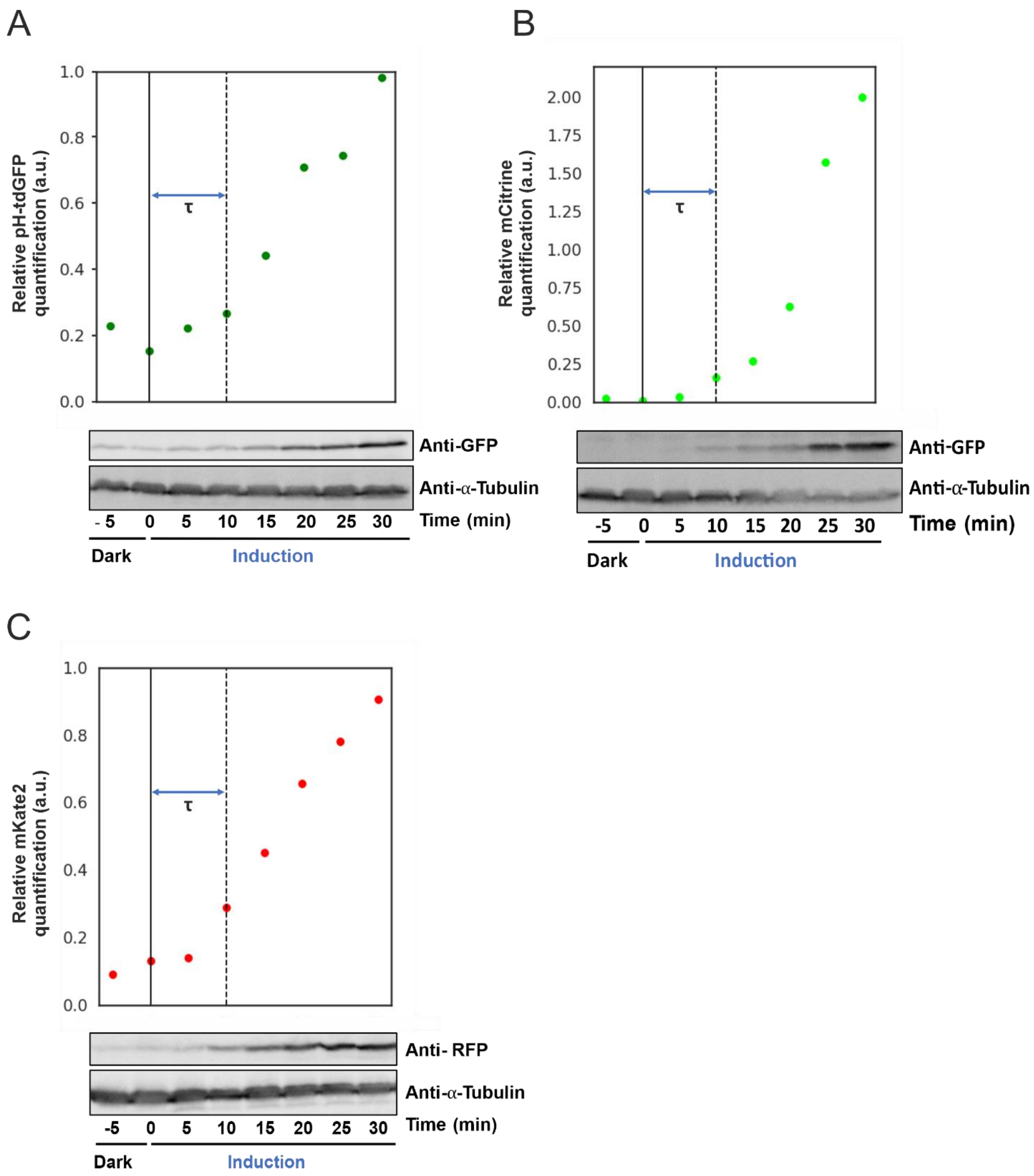

Figure S2 Estimation of total expression delay ($\tau_1 + \tau_2$) via Western Blot

A-C) Detection via Western Blot of the appearance of the fluorescent protein after the activation of the EL222_AQTrip system in cells expressing pHtdGFP (**A**), mCitrine (**B**) and mKate2 (**C**) under the control of EL222_AQTrip. The EL222_AQTrip system was activated at t=0. Measurements were carried out every 5 minutes. Relative abundance of the FPs was quantified as described in Methods. On the plots, τ indicates the first moment of a detectable increase in the relative abundance of the FPs indicating the upper bound for the total delay between the activation of the EL222_AQTrip system and the appearance of the FP ($\tau_1 + \tau_2 < \tau$). Independently of their sequences, the total delay for the FPs tested was between 5 and 10 minutes.

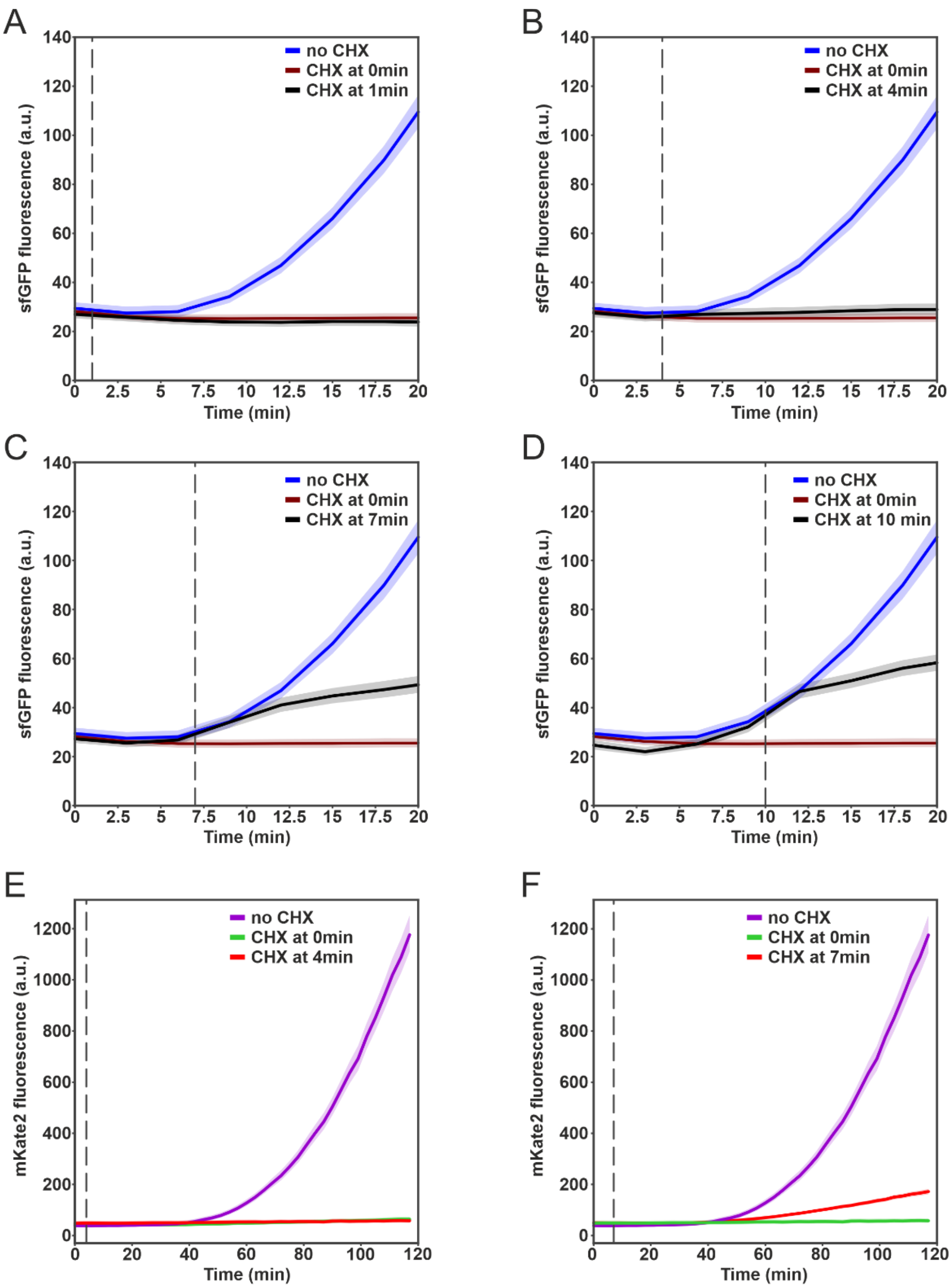

Figure S3. Estimation of total expression delay ($\tau_1 + \tau_2$) via fluorescence microscopy

A-D) Fluorescence increase in cells containing sfGFP under the control of the EL222_AQTrip system in response to light induction and subsequent addition of CHX. The EL222_AQTrip system was activated at time t=0. sfGFP imaging and EL222_AQTrip activation were carried out every 3 minutes. CHX (25 µg/ml final) was added at different time points after induction (vertical dashed lines). No CHX was added in the negative control (blue), while CHX was added at t=0 (just prior to induction) for the positive control (red). The appearance of mature FP (black) in panels C and D (CHX addition 7 and 10 minutes post-induction) indicates that immature protein had already been produced prior to CHX addition. On the other hand, no immature FP was present 1 and 4 minutes post-induction (Panels A and B). **E-F)** The same experiment repeated for mKate2, a very slow-maturing red FP. The appearance of mature FP on panel F (CHX added 7 min post-induction) and not on panel E (CHX added 4 min post-induction) shows that immature protein was produced between these two time points, in line with the sfGFP result of panels A-D.

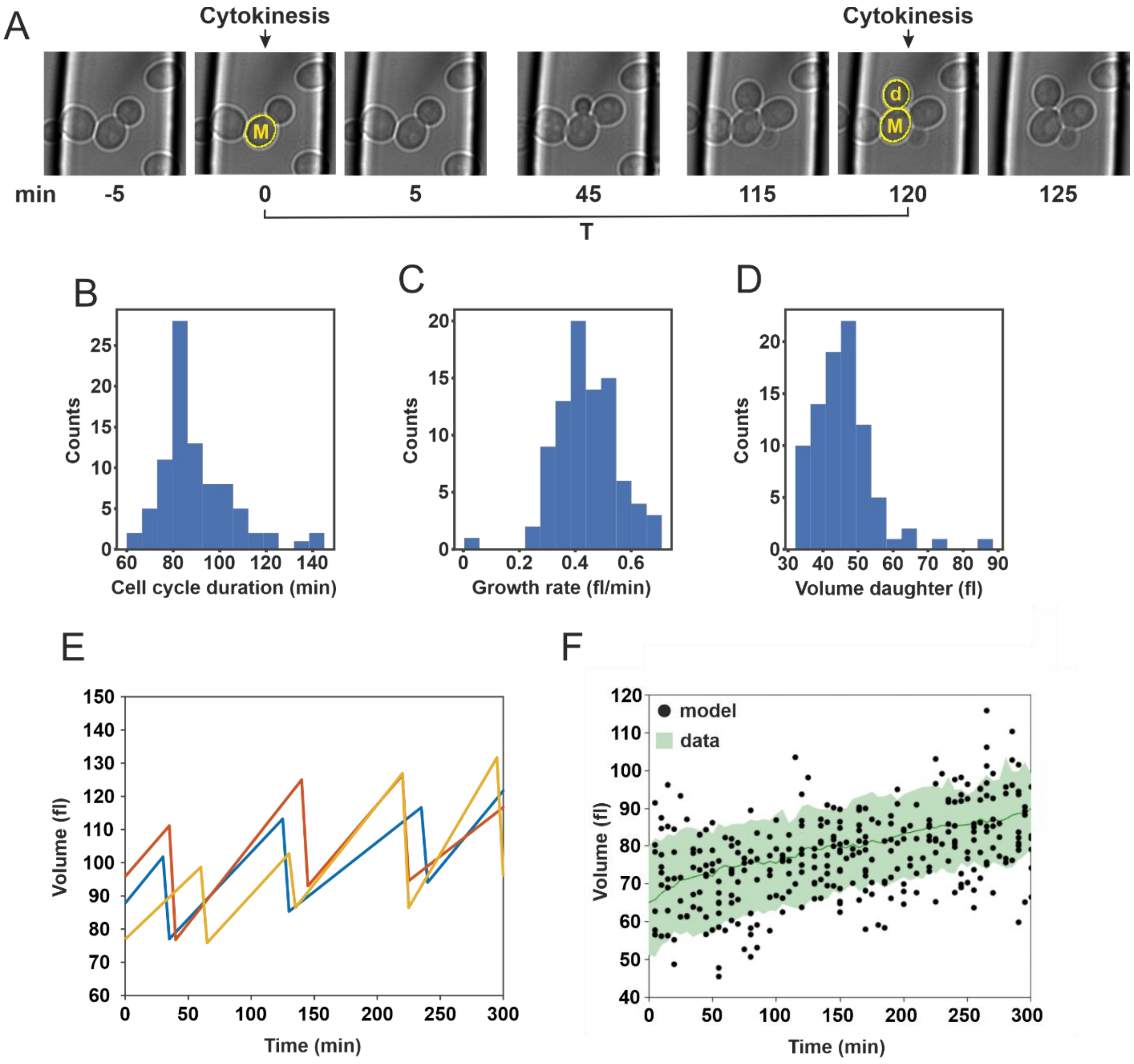

Figure S4

A) Example of the determination of the cell cycle parameters (T, μ, V_d). Cell cycle duration (T) was determined as the time between two cytokinesis events. Growth rate (μ) was obtained by subtracting the volume of the mother (M) at the beginning of a cell cycle (first cytokinesis event) from the combined volume of the mother (M) and the daughter (d) at the end of the same cell cycle (second cytokinesis event) and subsequently dividing by T. The volume lost at the end of the cell cycle (V_d) was defined as the volume of the daughter (d). Cytokinesis events were identified by the darkening of the bud neck and by the slight movement of the bud away from the mother cell. Cell volume was calculated based on the segmentation masks given by BudJ (yellow lines, see also Methods), by approximating the cell shape with an ellipsoid. **B-D)** Example of cell cycle-related parameter distributions for cells expressing mCitrine under the control of the EL222_AQTrip after light activation. Cell cycle duration (**B**), growth rate (**C**) and the volume of the bud at the end of the cell cycle (**D**) (n=87) were measured from the same cells for which the fluorescence increase was quantified (see Methods for details). These data were fitted with a multivariate log-normal distribution from which the cell cycle parameters of simulated cells were sampled (T_c, μ_c and $V_{d,c}$, see Methods for details). **E)** Example of volume dynamics for three simulated cells. Cell cycle parameters (T_c, μ_c and $V_{d,c}$) were sampled from the multivariate distribution derived from **B-D** (see Methods). **F)** Comparison of the volume dynamics of the mother cells analyzed for panels **A-C** (green bands extend from the 25th to the 75th percentile, n=47 cells) with the dynamics of a set of simulated cells (black dots, n=500). For the

simulated cells, the volume at the beginning of each cell cycle ($V_{0,c}$, see Methods) is plotted. Cell cycle parameters (T_c, μ_c and $V_{d,c}$) for the simulated cells were sampled from the multivariate distributions derived from **B-D**. The distribution of volumes at t=0 for the simulated cells was derived from the volume distribution at t=0 of the experimentally measured cells. The overall trend and variability in the volume dynamics are comparable between simulation and experiment, indicating that our volume model adequately captures the main features of growth and division. Note that mother yeast cells increase in size over successive divisions (12), and this trend is also captured by our model.

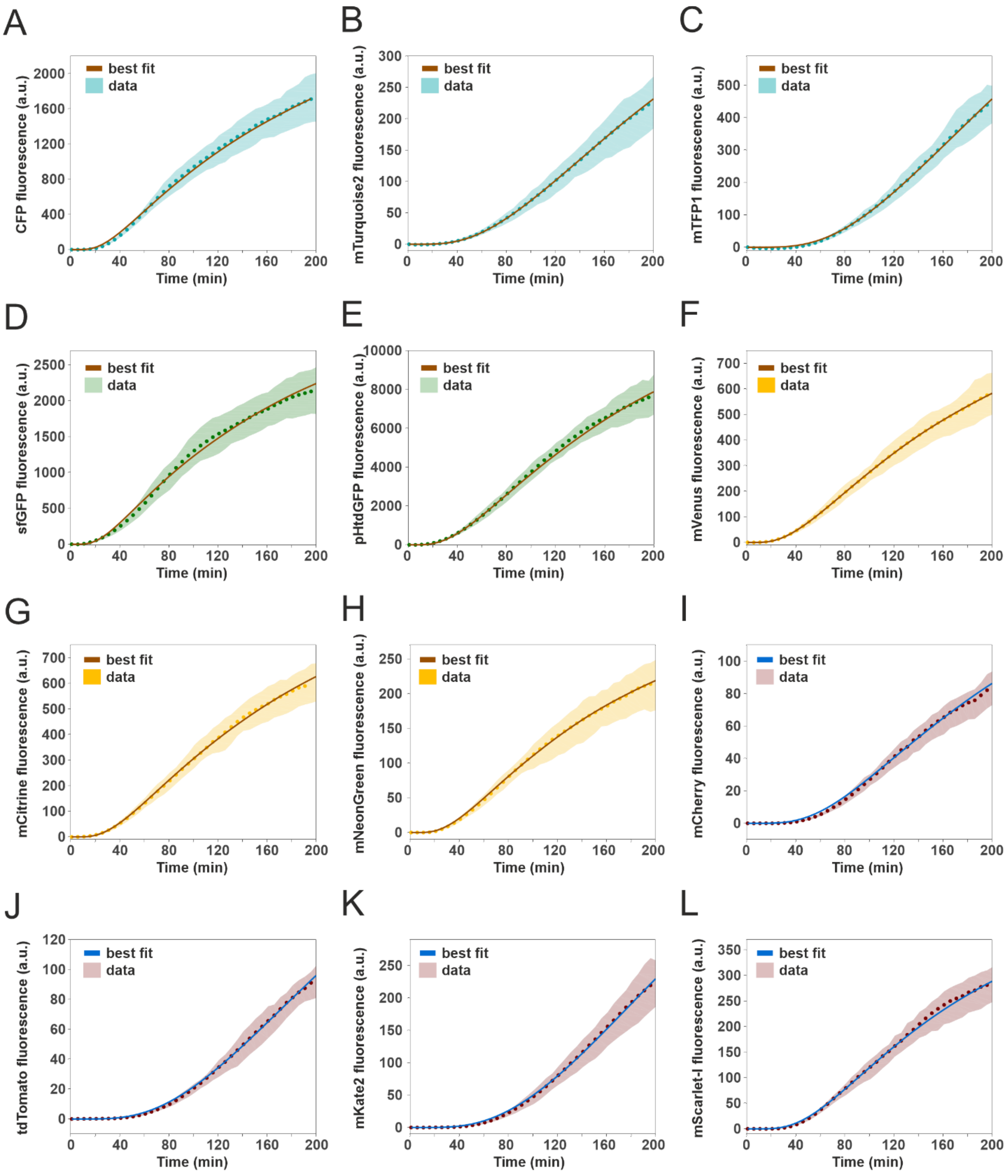

Figure S5

A-L) Comparison of experimentally measured fluorescence increase and the model best fit for cells expressing Cerulean, n= 60 (**A**), mTurquoise2 (n=62) (**B**), mTFP1 (n=48) (**C**), sfGFP (n=55) (**D**), pHtdGFP (n=57) (**E**), mVenus (n=48) (**F**), mCitrine (n=47) (**G**), mNeonGreen (n=50) (**H**) mCherry (n=52) (**I**), tdTomato (n=39) (**J**), mKate2 (n=53) (**K**) and mScarlet-I (n=44) (**L**) under the control of the EL222_AQTrip system. The EL222_AQTrip system was activated at t=0**.** Shaded bands extend from the 25th to the 75th percentile. Dots represent the mean. See Methods for detailed description of the model and the fitting procedure.

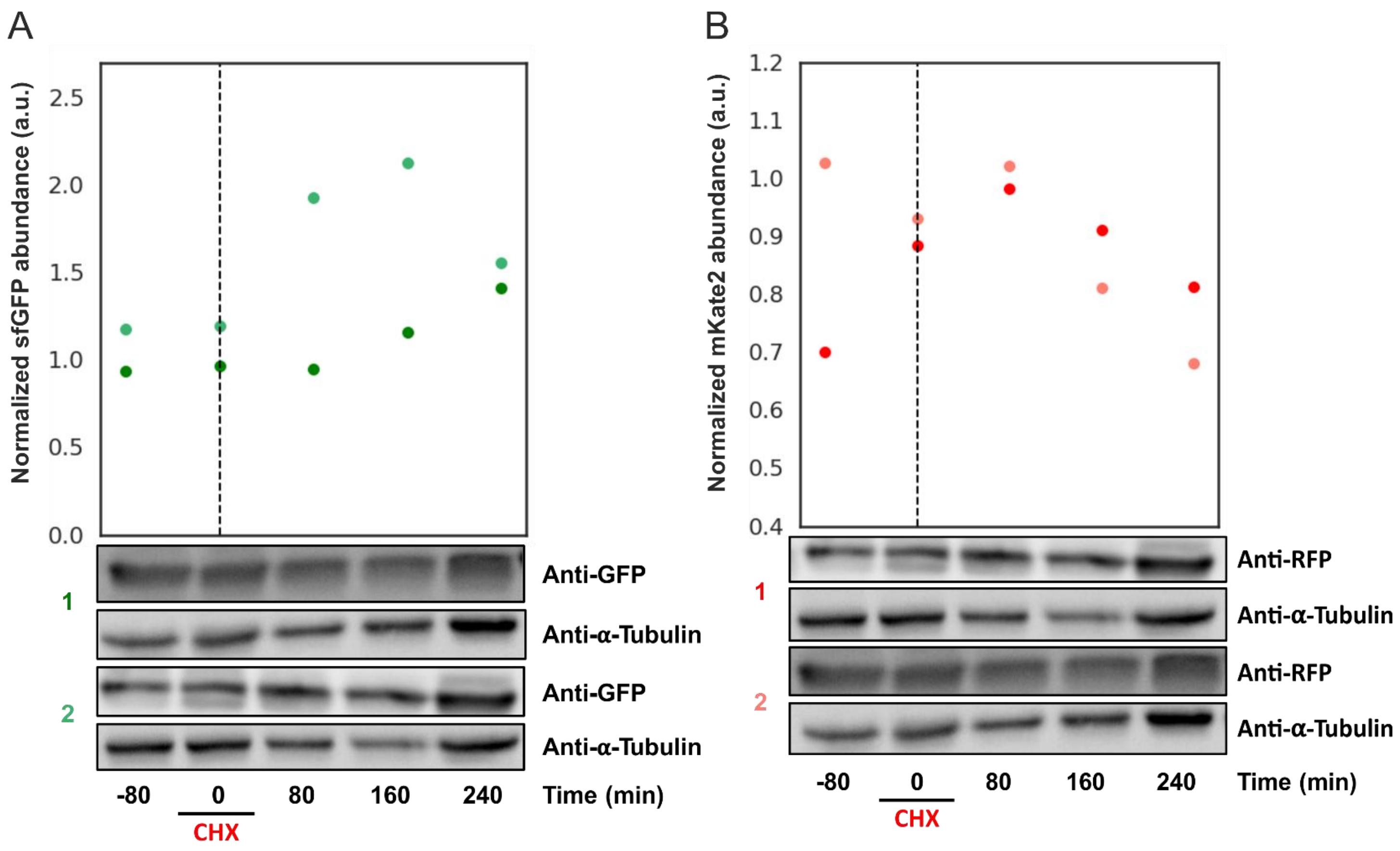

Figure S6: Western blot quantification of sfGFP and mKate2 abundance in response to CHX treatment

Given the observation that FP concentration seems to decrease after CHX addition (Fig. 2 of the main text), we tested whether this decrease could be attributed to protein degradation by quantifying FP abundance before and after CHX treatment. In this experiment, CHX was added to growing cells expressing sfGFP (panel A) or mKate2 (panel B) under the control of the constitutive TEF1 promoter. Experiments were carried out in technical duplicates (displayed with markers of different intensity), and FP abundance normalized to loading control was determined as described in the Methods section. No clear changes in FP abundance are observable after treatment, suggesting that the proteins are not degraded.

Supplementary Notes

Supplementary Note 1: Optimization of the induction of the EL222_AQTrip system

To minimize the effects of phototoxicity due to repeated stimulation of EL222_AQTrip, and reduce FP photobleaching, we tested several patterns of light excitation in order to achieve maximal FP induction while minimizing the amount of light that the cell received. We carried out our tests on a strain containing mKate2 under the control of the EL222-AQTrip system. Using an FP that is excited with wavelengths outside the absorption spectrum of the LOV domain (13), allowed us to separate the effect of light stimulation on EL222 activation from stimulation of EL222 due to excitation light used for FP imaging.

Given the very slow dark-reversion of the EL222_AQTrip, we expected that it could be fully activated by intermittent light exposures. We first tested a pulsed light setting used to image cyan FPs, which consisted of short (300ms) light pulses at 440nm (1400mW/cm^2) every 5 min (Fig. S1C). According to previous tests (14), the amount of light received by the cells in this setting should fully activate EL222_AQTrip. Reducing this amount by increasing the spacing between the pulses (300ms at 1400mW/cm^2 every 10 minutes) or by decreasing the intensity of the pulses (1s at 100mW/cm^2 every 5 minutes) achieved the same fluorescence dynamics (Fig. S1C), indicating that the system is fully activated by all the light settings tested. We therefore chose the setting with the lowest light intensity (1s at 100mW/cm^2 every 5 minutes) for carrying out our experiments. In this setting, the amount of light received by the cells is smaller than what is normally used for fluorescence imaging, and the average doubling time of mother cells exposed to blue light is indistinguishable from the doubling time of mothers in this strain background (Fig. S4A). Combining light stimulation with FP imaging every 5 minutes provided sufficiently high temporal resolution for fluorescence measurements and sufficient time to activate and image an adequate number of cells over different XY positions. Note that imaging cyan and green fluorescent proteins (excitation wavelengths around 440nm and 470nm respectively) also activates the EL222-AQTrip system, but this additional exposure does not produce higher induction since the system already operates at saturation.

Supplementary Note 2: Estimation of the total expression delay ($\tau_1 + \tau_2$)

To estimate the total delay between the activation of EL222_AQTrip and the appearance of the first translated immature fluorescent proteins ($\tau_1 + \tau_2$ in our model), we first used Western blotting to locate the moment of appearance of immature protein following light stimulation. In order to evaluate potential differences in expression dynamics due to the FP sequence differences, we tested three different strains expressing respectively a green (pHtdGFP), a yellow-green (mCitrine) and a red (mKate2) fluorescent protein upon activation of the EL222 optogenetic system. As Fig. S2A-C shows, immature FP appears at some point between 5 and 10 minutes after light induction, independently of the FP tested.

To validate these findings and obtain a more precise estimate of the total expression delay ($\tau_1 + \tau_2$), we used time-lapse microscopy with a time resolution of 3 min. Using a strain carrying sfGFP under the control of the EL222-AQTrip system, we measured the fluorescence increase after the addition of CHX at different time points after the start of light stimulation (Methods). CHX blocks the translation of new immature FPs. Therefore, if CHX is added before any immature protein has been translated (i.e. earlier than $\tau_1 + \tau_2$ minutes post-induction), we would expect to see no increase in fluorescence. On the other hand, if CHX is added at a later point, after some immature FP has been translated (i.e. after $\tau_1 + \tau_2$ minutes post-induction), we would expect those immature proteins to mature and observe an increase in the fluorescence signal. We correctly observed no fluorescence increase in cells treated with CHX prior to light stimulation, and the same observation was made for cells treated with CHX after 1 or 4 minutes following light stimulation (Fig.S3A,B). On the other hand, we observed a clear increase in fluorescence in cells treated with CHX after 7 or 10 minutes after the start of light stimulation (Fig.S3C,D). These results suggest that the first translated sfGFP molecules appear between 4 and 7 minutes after the light stimulation, in good agreement

with the results obtained via Western blot. To further confirm that the total expression delay is not dependent on the FP chosen we repeated the same experiment using a strain carrying mKate2 under the control of the EL222-AQTrip system (Fig.S3E,F). This experiment gave similar results indicating that also for mKate2 the first translated molecules appear between 4 and 7 minutes after the light induction.

Given the Western blot and CHX results, we set the total expression delay ($\tau_1 + \tau_2$) at 6 minutes.

Supplementary Note 3: Mathematical modeling of histone synthesis and histone-FP fluorescence dynamics

Histones are synthesized during the S phase of the cell cycle, when DNA is replicated. Histone expression during the cell cycle can be followed by creating a fusion of a histone and a fluorescent protein (H-FP fusion). To observe how the FP maturation dynamics can affect the observed histone expression dynamics in dividing mother cells, we devised a simple mathematical model that accounts for histone expression, FP maturation and cell division. The model describes the dynamics of the H-FP fusion between two divisions of a mother cell, taking into account the fact that half of the histones are lost each time a bud separates from the mother cell, since the newborn daughter receives one genome copy and the associated histones. The divisions are separated by $T_d = 100$ min, which is the average division time of our Hta2-mRFP1 strains in glucose. DNA replication starts at the moment of bud emergence ($t_{bud} = 43$ min on average in our experiments) and ends 40 min later ($t_{end} = 83$min estimated from previous work (15)) (Fig.2C). Given that histone expression tightly follows DNA replication (16,17), it can be safely assumed that the H-FP fusion is only expressed during this interval and that the rate of histone synthesis has the form

$$k_{p,H}(t) = \begin{cases} 0 & 0 \leq t < t_{bud} \\ r, & t_{bud} \leq 1 < t_{end} \\ 0, & t_{end} \leq t \leq T_d \end{cases}$$

For simplicity, we further assumed that histones are synthesized at a constant rate r, and that there is no histone degradation. Therefore, the histone abundance, P_H, at division ($t = T_d$) is given by

$$P_H(T_d) = P_H(0) + r \cdot (t_{end} - t_{bud})$$

where $t = 0$ denotes the moment of the previous division. Given that histone abundance needs to double during each cell cycle, $P_H(T_d)$ needs to equal $2P_H(0)$, which means that $r \cdot (t_{end} - t_{bud}) = P_H(0)$, or $r = P_H(0)/(t_{end} - t_{bud})$. Therefore, choosing an initial condition $P_H(0) = 1$ (in arbitrary units), we calculated the value of r needed for a doubling of histone abundance within the period of DNA replication.

To take into account the presence of immature FP with a single maturation step, we defined two protein species, HP_d and HP_m, corresponding to the histone fusion to the immature (dark) and mature (fluorescent) FP forms respectively. The synthesis rate $k_{p,H}(t)$ defined above was used to simulate the synthesis of HP_d, while HP_m was obtained by maturation of HP_d, according to the one-step maturation model already described:

$$\frac{dHP_d}{dt} = k_{p,H}(t) - k_m HP_d$$
$$\frac{dHP_m}{dt} = k_m HP_d$$

Contrary to the histone abundance P_H described above, the abundances $HP_d(0)$ and $HP_m(0)$ at the start of a cell cycle are unknown, because immature FP from the previous cell cycle is still maturing into the next. We therefore obtained the appropriate starting abundances using the iterative simulation of a large number (30) of cell cycles. The first cycle was started with $HP_d(0) = 1$ and $HP_m(0) = 0$. At each subsequent division ($t = T_d$), $HP_d(T_d)$ and $HP_m(T_d)$ were divided in half, and a new cycle was simulated with $HP_d(0) = 0.5 \cdot HP_d(T_d)$ and $HP_m(0) = 0.5 \cdot HP_m(T_d)$. At the end of these iterations, the initial abundances of HP_d and HP_m had stabilized to their steady-state values, and were used for our plots.

For two-step FP maturation, the model was defined by

$$\frac{dHP_{d1}}{dt} = k_{p,H}(t) - k_{m1}HP_{d1}$$
$$\frac{dHP_{d2}}{dt} = k_{m1}HP_{d1} - k_{m2}HP_{d2}$$
$$\frac{dHP_m}{dt} = k_{m2}HP_d$$

and the appropriate initial conditions for $HP_{d1}(0)$, $HP_{d2}(0)$ and $HP_m(0)$ were obtained by the same iterative scheme described above.

Supplementary Note 4: Selection between 1-step and 2-step maturation models for cyan and red FPs

Fitting a one-step maturation model (M_1) to some expression FP profiles resulted in systematic deviations from the experimental data (Y), which suggested the presence of more complex maturation kinetics. Such deviations were not observed for any of the green and yellow-green proteins that we tested, but were apparent for some of the cyan (mTurquoise2, mTFP1) and all red FPs. Fitting a two-step model (M_2) resulted in improved fits to the experimental data, which suggested that a two-step model is a better choice. This could also be verified by calculating the Akaike Information Criterion (AIC) (18) for M_1 and M_2 and comparing the AIC values. The AIC for a given model structure (one/two-step) is defined as

$$\mathrm{AIC} = -2\ln(L(Y|\theta^*)) + 2K,$$

where $L(Y|\theta^*)$ is the likelihood function evaluated at θ^*, the parameter vector that maximizes the likelihood, and K is the number of free parameters in the model. In the case of normally distributed measurement noise that we consider here, the term $-2\ln(L(Y|\theta^*))$ is equal to $\chi^2(\theta^*)$, which is the sum of squared deviations of the model predictions from the experimental data, weighted by the measurement noise variance (cf. Methods for the precise definition of $\chi^2(\theta)$). For both one- and two-step models, $K = 3$, which is the number of free (identifiable) parameters (note that we consider two-step models with $k_{m1} = k_{m2}$, which reduces the number of free parameters by one in the two-step model).

Putting everything together, the difference in the AIC values between M_1 and M_2, $\mathrm{AIC}_2 - \mathrm{AIC}_1$, is equal to $\chi_2^2(\theta_2^*) - \chi_1^2(\theta_1^*)$, the difference between the sum of weighted squared deviations. Since $\chi_2^2(\theta_2^*)$ was always smaller than $\chi_1^2(\theta_1^*)$ in the models that we tested, the two-step model had a smaller AIC than the one-step model (i.e. $\mathrm{AIC}_2 < \mathrm{AIC}_1$). The probabilities that M_1 or M_2 is the best model given the experimental data are then given by (18)

$$p_1 = \frac{\exp(-\mathrm{AIC}_1 + \mathrm{AIC}_2)}{1 + \exp(-\mathrm{AIC}_1 + \mathrm{AIC}_2)} \text{ and } p_2 = \frac{1}{1 + \exp(-\mathrm{AIC}_1 + \mathrm{AIC}_2)}$$

The table below summarizes the AIC values for M_1 and M_2 for the cyan and red FPs that were fitted with both models. As can be seen, the weight of evidence in favor of the two-step model (given by p_2) is overwhelming in all cases.

FP	AIC_1	AIC_2	p_1	p_2
mTurquoise2	3.15	0.41	0.06	0.94
mTFP1	31.94	4.96	1.9e-12	≈1
mScarlet-I	21.29	10.6	2.2e-5	≈1
mCherry	50.37	19.03	2.4e-14	≈1
tdTomato	78.07	8.14	4.2e-31	≈1
mKate2	147.65	6.49	4.9e-62	≈1

Supplementary Note 5: Identifiability analysis of the abundance models

Local identifiability of one- and two-step models

We consider models (1a-c) or (2a-d) without the time-delay terms. Given the fact that our models are built from the cascade interconnection of linear modules with time-delayed inputs, the outputs of the delayed and non-delayed systems should be identical, except for the fact that the former will be delayed by $\tau_1 + \tau_2$. Therefore, the identifiability properties of the non-delayed models carry over to the delayed models.

We will rewrite our one-step (delay-free) maturation model using matrix-vector notation as follows:

$$\dot{x} = Ax, \;\; x(0) = x_0 \quad (1a)$$
$$y = Cx \quad (1b)$$

x has the form $x = [u \;\; m \;\; P_d \;\; P_m]^T$, where T denotes the matrix transpose and u denotes an “input” state with zero dynamics and initial condition $u(0) = 1$, which is used to model the constant light input applied to the system. All other entries of $x(0)$ are zero, so that $x_0 = [1 \;\; 0 \;\; 0 \;\; 0]^T$. Matrix A has the form

$$A = \begin{bmatrix} 0 & 0 & 0 & 0 \\ k_r & -k_{dr} & 0 & 0 \\ 0 & k_p & -k_m & 0 \\ 0 & 0 & k_m & 0 \end{bmatrix}$$

and $C = [0 \;\; 0 \;\; 0 \;\; 1]$. We next define the vector θ which contains all unknown parameters in matrix A (i.e. $\theta = [k_r \;\; k_{dr} \;\; k_p \;\; k_m]$). In this way, we can treat A as a function of θ and write it as $A(\theta)$. We then consider the function

$$N(\theta) = [Cx_0 \;\; CA(\theta)x_0 \;\; CA^2(\theta)x_0 \; \ldots \; CA^7(\theta)x_0 \,]$$

following the definitions of (19). The system will then be *locally identifiable* at a point θ_0 if the Jacobian of $N(\theta)$ has rank 4 (i.e. full column rank) at θ_0, implying that the map $N(\theta)$ is injective at θ_0 (19).

Performing a symbolic calculation of the Jacobian at an arbitrary (non-zero) point θ_0, we obtain a column rank of 3, which suggests that not all parameters are identifiable. To investigate the source of this lack of identifiability, we turn to the analytical solution of (1a,b). Using results from linear systems theory (20), we get

$$m(t) = \frac{k_r}{k_{dr}}(1 - \exp(-k_{dr}t))$$
$$P_d(t) = \frac{k_r k_p}{k_{dr}k_m}(1 - \exp(-k_m t)) + \frac{k_r k_p}{k_{dr}}\left(\frac{\exp(-k_m t) - \exp(-k_{dr}t)}{k_m - k_{dr}}\right)$$

The expression for $P_m(t)$ (the measured output) is then obtained by $\int_0^t k_m P_d(s)ds$. However, carrying out the integration is not necessary, as long as we observe that the factor $k_r k_p$ will appear in all of the terms making up $P_m(t)$. Knowing that k_r and k_p always appear in a product in the expression of $P_m(t)$, the lack of identifiability can be explained by the fact that only the *product* $k_r k_p$ can be identifiable. Fixing k_r at 1 can done without loss of generality, and in that case the Jacobian of $N(\theta)$ has full column rank of 3, which implies that k_{dr}, k_p and k_m are locally identifiable at any non-zero θ_0 when $k_r = 1$. This result in turn implies that k_m (the parameter of interest) can in principle be uniquely identified in the neighborhood of a ground-truth parameter k_{m0} (further information on the different identifiability definitions can be found in (21)).

For the two-step maturation model, we can apply the same approach as above to the system with

$$A = \begin{bmatrix} 0 & 0 & 0 & 0 & 0 \\ k_r & -k_{dr} & 0 & 0 & 0 \\ 0 & k_p & -k_{m1} & 0 & 0 \\ 0 & 0 & k_{m1} & -k_{m2} & 0 \\ 0 & 0 & 0 & k_{m2} & 0 \end{bmatrix}, \; C = [0 \quad 0 \quad 0 \quad 0 \quad 1] \text{ and } x_0 = [1 \quad 0 \quad 0 \quad 0 \quad 0]^T.$$

Resolving the non-identifiability of k_r and k_p by fixing $k_r = 1$ results in $N(\theta)$ with column rank of 4, which implies that k_{dr}, k_p, k_{m1} and k_{m2} are locally identifiable. Therefore, the two parameters of interest (k_{m1} and k_{m2}) can in principle be uniquely locally identified.

An observation on the maturation half-time of two-step models

Two-step maturation models contain two maturation rates (k_{m1} and k_{m2}), and a maturation half-time can be associated with each step, i.e. $t_{1,50} = \ln(2)/k_{m1}$ and $t_{2,50} = \ln(2)/k_{m2}$. However, the overall maturation half-time (t_{50}) of an FP with a two-step mechanism is *not* equal to $t_{1,50} + t_{2,50}$. Instead, this sum is always a *lower bound* on t_{50}. To see this, we can simulate the simplified two-step model

$$\begin{aligned} \dot{P}_{d1} &= -k_{m1}P_{d1} \\ \dot{P}_{d2} &= k_{m1}P_{d1} - k_{m2}P_{d2} \\ \dot{P}_m &= k_{m2}P_{d2} \end{aligned}$$

with the initial condition $P_{d1}(0) = 1$, $P_{d2}(0) = P_m(0) = 0$, i.e. assuming that we start with a pool of immature precursor P_{d1}. Scanning over a large range of $t_{1,50}$ and $t_{2,50}$ values by varying k_{m1} and k_{m2}, we can then plot the difference between the maturation half-time of this model (i.e. the time point t_{50} at which $P_m(t_{50}) = 0.5$) and the sum $t_{1,50} + t_{2,50}$, divided by $t_{1,50} + t_{2,50}$ (Fig. 1).

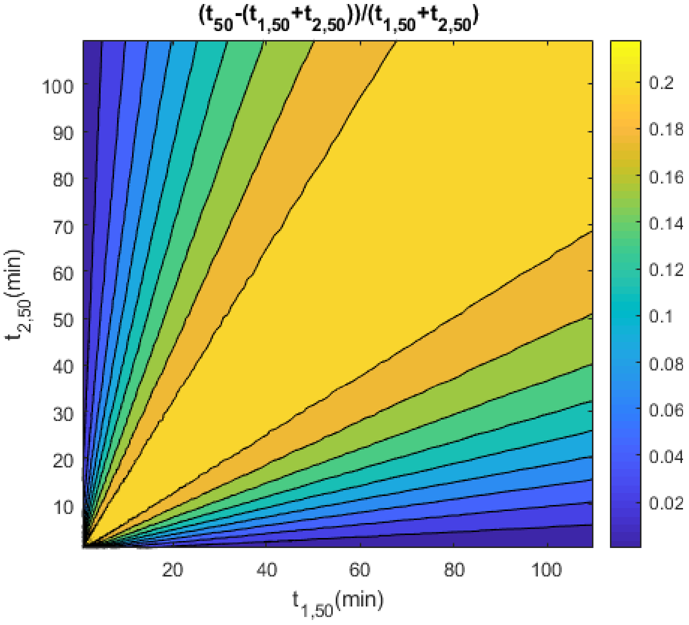

Figure 1

As Fig. 1 shows, the percentage by which t_{50} exceeds $t_{1,50} + t_{2,50}$ varies from 0 (when one maturation half-time tends to zero, i.e. one of the two steps becomes infinitely fast) up to around 24%. Therefore, the maturation half-time of the two-step model (t_{50}) is always lower-bounded by $t_{1,50} + t_{2,50}$; that is, $t_{50} \geq t_{1,50} + t_{2,50}$. Unfortunately, obtaining an analytical formula for t_{50} in terms of k_{m1} and k_{m2} was not impossible, and t_{50} could only be obtained via simulation.

Practical identifiability of two-step models

While the results of the local analysis suggest that k_{m1} and k_{m2} can be uniquely locally identified, in practice it is challenging to determine their values with good accuracy. This is because k_{m1} and k_{m2} and compensate for each other to some degree, which results in small changes in the observed output (the fluorescent protein). This result can be inferred by examining the qualitative properties of the two-step model. Besides Fig. 1, which provides an indication on how the two-step model solution differs from a simple exponential solution that uses the sum of k_{m1} and k_{m2} in a single maturation step, we can also plot the maturation half-time of the two-step model as k_{m1} and k_{m2} vary:

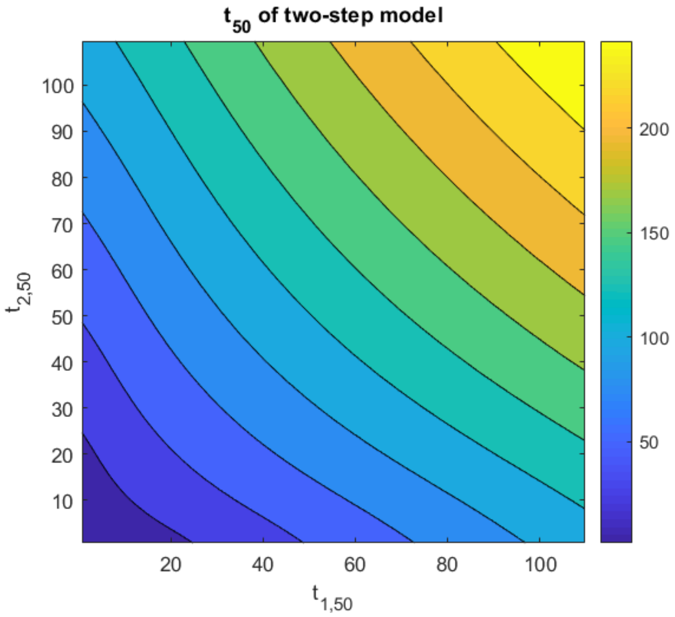

Figure 2. Maturation half-time of the two-step model as a function of the individual step half-times

Combining the information from Fig. 1 (the shape of the two-step solution) with the information from Fig. 2 (the dominant timescale of the two-step solution), we can see that, for a given maturation half-time (a contour curve in Fig. 2), the shape of the model solution (Fig. 1) remains relatively unchanged for a large range of k_{m1} and k_{m2} values, especially if k_{m1} and k_{m2} do not differ too much (i.e. in the cases when a two-step model is most relevant). Therefore, it can be anticipated that individual estimation of k_{m1} and k_{m2} will be difficult in practice. Furthermore, individual estimates of k_{m1} and k_{m2} do not seem to be particularly relevant for the model behavior. Instead of pinpointing the exact values of k_{m1} and k_{m2}, one can therefore focus on identifying a *common* value for both maturation rates, which results in a dominant time scale that matches the experimental data.

Supplementary Note 6: a simplified concentration model

The increase of volume in the bud attached to a mother cell implies that the FP content of the mother-bud system will be diluted in the absence of protein synthesis, leading to a reduction in FP concentration. Given that FP concentration is the same in the mother cell and the bud, it will not change at division. FP abundance on the other hand does change, and this is the reason why we track bud and mother volumes at division in our model (cf. Eq. (5) of Methods). This approach has the advantage that it can account for population variability in growth rates and cell cycle durations, as well as for the fact that mother cells gradually increase in volume over successive divisions (12) (cf. also Fig. S4E). At the same time, it increases the overall computational cost model runs, which in turn increase the runtime of parameter estimation and profile likelihood estimation.

As an alternative to the complex model described in the main text, one could consider a simpler model in which dilution due to bud growth is captured by a linear term that describes the average effect of dilution across the monitored cell population. More concretely, for one-step FP maturation, we can consider a model of the form

$$\dot{m}_c = k_r s(t - \tau_1) - k_{dr} m_c - k_{dil} m_c$$

$$\dot{P}_{d,c} = k_p m(t - \tau_2) - k_m P_{d,c} - k_{dil} P_{d,c}$$
$$\dot{P}_{m,c} = k_m P_{d,c} - k_{dil} P_{m,c}$$

where k_{dil} denotes an equivalent average dilution rate across the monitored cell population, and the state variables ($m_c, P_{d,c}$ and $P_{m,c}$) denote concentrations of the respective species (note that, in a strict sense, the interpretation of the synthesis rates k_r and k_p is different in this model compared to the abundance model used in the main text, but we use the same symbols to simplify notation. Moreover, we still need to set $k_r = 1$ to avoid the identifiability problem described in Supplementary Note 5).

Since we are monitoring a fixed number of mother cells, dilution arises from the growth of buds. We can therefore calculate k_{dil} based on the same single-cell data as above (Fig. S4). To do this, we need the average cell cycle duration of mother cells ($\bar{T}_c$), and the average fraction of newborn daughter cell volume to the mother volume just prior to division (r) (cf. Fig. S4 and Methods). After a division, the volume of a mother cell will increase by a factor $1 + r$ until the next division, which will happen $\bar{T}_c$ minutes later. Therefore, assuming constant abundance P of a given species, the concentration of P just after a division (denoted $P_c(0)$) will decrease by a factor $1/(1 + r)$ until the next division. Assuming exponential volume increase, the concentration of P at time t will be given by $P_c(t) = P_c(0)\exp(-k_{dil}t)$. After $\bar{T}_c$ minutes, $P_c(\bar{T}_c)$ will be equal to $1/(1 + r)P_c(0)$. Based on these data, we obtain the equivalent dilution rate $k_{dil} = \log(1 + r)/\bar{T}_c$.

As can be verified by repeating the calculations of Supplementary Note 5, the concentration model written above remains locally identifiable when k_{dil} is fixed (i.e. not estimated from data). Therefore, this model can be fitted to the average mature FP concentration data to estimate the maturation rate k_m. Concentration models for two-step maturation kinetics can be handled in the same manner, and are not described explicitly.

Fitting the simplified concentration model to our FP data, we obtained maturation half-times that were similar to those obtained with the full model (with the exception of mTurquoise2 and mVenus, which showed greater deviations):

Protein	Half-time simple model (min)	Half-time full model (min)
CFP	8.3	9.7
mCherry	20.4	21.6
mCitrine	11.8	10.4
mKate2	47.5	53.5
mNeonGreen	10.9	11.6
mScareltI	12.0	12.9
mTurquoise2	19.5	27.5
mTFP1	33.6	31.6
mVenus	13.8	20.8
pH-tdGFP	12.3	13.7
sfGFP	6.9	6.9
tdTomato	42.2	38.4

It should be noted that, although the simplified model can be simulated at a fraction of the time required for the more complex model used in the main text, it still requires single-cell data to estimate r and $\bar{T}_c$, which are required for the calculation of k_{dil}. Moreover, the simplified model is based on the assumption that the average dilution rate stays constant across an experiment, an assumption which is not entirely correct given the observed mother volume dynamics: as can be seen on Fig. S4, mother cell volume increases over time as cells age, and thus the ratio of daughter to mother volume changes as well. Still, our results above suggest that the simplified model is a viable alternative to the more complex model used in the main text, especially when computational resources are limited.

Supplementary Note 7: maturation rate sensitivity to the total expression delay $(\tau_1 + \tau_2)$

Given that the total expression delay ($\tau_1 + \tau_2$) may vary slightly across different FPs, while some degree of uncertainty in the experimental determination of this delay is always inevitable, it is instructive to inspect how the maturation half-time estimates for different FPs change as the total delay varies. Fitting our model to FP concentration data using different assumed values for $\tau_1 + \tau_2$ produced the following results for the maturation half-time (red denotes the results obtained for $\tau_1 + \tau_2 = 6\,min$, which we obtained from the experiments described in Supp. Notes 2 and 3):

**Fluorescent protein**	**sfGFP**	**mNeonGreen**	**mVenus**	**mCherry**	**mKate2**
**τ_1+τ_2 (min)**	**t_{50} estimate (min)**				
3 min	8.7	11.2	21.2	23.1, 23.1	55.6, 55.6
4 min	9	12.5	18.8	22.1, 22.1	56.8, 56.8
**6 min**	**6.9**	**11.6**	**20.8**	**21.6, 21.6**	**53.5, 53.5**
8 min	3.9	12.6	21.8	18.3, 18.3	44.9, 44.9
10 min	6.5	6.9	21.9	17.2, 17.2	44.9, 44.9
15 min	3.5	6.1	15.3	14.8, 14.8	33.1, 33.1
20 min	3.5	3.5	3.8	13.7, 13.7	28.9, 28.9

As can be seen, the estimated maturation half-time of fast-maturing FPs (sfGFP, mNeonGreen) is more sensitive to $\tau_1 + \tau_2$ in comparison to slower FPs such as mVenus, mCherry and mKate2. This is expected, since the value of the time delay affects the model prediction on the initial rise in fluorescence, and this rise is faster and sharper for fast-maturing FPs. For these proteins, values of $\tau_1 + \tau_2$ larger than 8-10 min result in t_{50} estimates that "jump" unpredictably as the model tries (and fails) to properly capture the fast initial rise in fluorescence (note also that 3.5 min is the smallest value of t_{50} allowed in our optimization runs). However, such values of $\tau_1 + \tau_2$ are anyway incompatible with the experimental results that we obtained with different FPs (sfGFP, mCitrine, mKate2), as described in Supp. Notes 2 and 3.

References

1. Benzinger D, Khammash M. Pulsatile inputs achieve tunable attenuation of gene expression variability and graded multi-gene regulation. Nat Commun. 2018;9(1).

2. Rullan M, Benzinger D, Schmidt GW, Milias-Argeitis A, Khammash M. An Optogenetic Platform for Real-Time, Single-Cell Interrogation of Stochastic Transcriptional Regulation. Mol Cell. 2018;70(4):745-756.e6.

3. Zhou X, Li S, Zhang J. Tracking the Activity of mTORC1 in Living Cells Using Genetically Encoded FRET-based Biosensor TORCAR. 2016;8:225–33.

4. Botman D, O'Toole TG, Goedhart J, Bruggeman FJ, van Heerden JH, Teusink B. A yeast FRET biosensor enlightens cAMP signalling. Mol Biol Cell. 2021 Apr 21;mbc.E20-05-0319.

5. San Martín A, Ceballo S, Baeza-Lehnert F, Lerchundi R, Valdebenito R, Contreras-Baeza Y, et al. Imaging mitochondrial flux in single cells with a FRET sensor for pyruvate. PLoS One. 2014;9(1).

6. Khmelinskii A, Keller PJ, Bartosik A, Meurer M, Barry JD, Mardin BR, et al. Tandem fluorescent protein timers for in vivo analysis of protein dynamics. Nat Biotechnol. 2012;30(7):708–14.

7. Roberts TM, Rudolf F, Meyer A, Pellaux R, Whitehead E, Panke S, et al. Identification and Characterisation of a pH-stable GFP. Sci Rep. 2016 Jun 21;6(1):1–9.

8. Nagai T, Ibata K, Park ES, Kubota M, Mikoshiba K, Miyawaki A. A variant of yellow fluorescent protein with fast and efficient maturation for cell-biological applications. Nat Biotechnol. 2002;20(1):87–90.

9. Shaner NC, Lambert GG, Chammas A, Ni Y, Cranfill PJ, Baird MA, et al. A bright monomeric green fluorescent protein derived from Branchiostoma lanceolatum. Nat Methods. 2013;10(5):407–9.

10. Liang JC, Chang AL, Kennedy AB, Smolke CD. A high-throughput, quantitative cell-based screen for efficient tailoring of RNA device activity. Nucleic Acids Res. 2012;40(20):1–14.

11. Zoltowski BD, Motta-Mena LB, Gardner KH. Blue light-induced dimerization of a bacterial LOV-HTH DNA-binding protein. Biochemistry. 2013;52(38):6653–61.

12. Janssens GE, Veenhoff LM. The natural variation in lifespans of single yeast cells is related to variation in cell size, ribosomal protein, and division time. PLoS One. 2016;11(12):1–18.

13. Nash AI, McNulty R, Shillito ME, Swartz TE, Bogomolni RA, Luecke H, et al. Structural basis of photosensitivity in a bacterial light-oxygen-voltage/helix-turn-helix (LOV-HTH) DNA-binding protein. Proc Natl Acad Sci. 2011;108(23):9449–54.

14. Benzinger D, Ovinnikov S, Khammash M. Synthetic gene networks recapitulate dynamic signal decoding and differential gene expression. bioRxiv. 2021 Jan 1;2021.01.07.425755.

15. Garmendia-Torres C, Tassy O, Matifas A, Molina N, Charvin G. Multiple inputs ensure yeast cell size homeostasis during cell cycle progression. Elife. 2018;7:1–27.

16. Gunjan A, Paik J, Verreault A. Regulation of histone synthesis and nucleosome assembly.

Biochimie. 2005;87(7):625–35.

17. Nelson DM, Ye X, Hall C, Santos H, Ma T, Kao GD, et al. Coupling of DNA Synthesis and Histone Synthesis in S Phase Independent of Cyclin/cdk2 Activity. Mol Cell Biol. 2002;22(21):7459–72.

18. Portet S. A primer on model selection using the Akaike Information Criterion. Infect Dis Model. 2020;5:111–28.

19. Van Den Hof JM. Structural identifiability of linear compartmental systems. IEEE Trans Automat Contr. 1998;43(6):800–18.

20. Chen C-T, Shafai B. Linear system theory and design. Oxford University Press; 1999.

21. Walter E, Pronzato L. Identification of parametric models: from experimental data. Springer Verlag; 1997.
