# Supplementary material for: Systematic In Vivo Characterization of Fluorescent Protein Maturation in Budding Yeast
Source: ACS Synth Biol. 2022 Feb 18;11(3):1129–41. doi: 10.1021/acssynbio.1c00387 (PMC8938947; doi:10.1021/acssynbio.1c00387)
Supplement: Supplementary file 2 — sb1c00387_si_002.zip [file sb1c00387_si_002.zip › Supplementary Data/Info.docx]

Each of the accompanying folders contains the following data for each of the FPs considered in this work:

1. The log-mean vector and the log-covariance matrices of the multivariate log-normal distributions fitted to the three cell cycle parameter distributions (T, µ and V_d_ (see Methods)) (files: GDmeanLL.csv and GDcovLL.csv respectively)
2. The mean and the 25^th^ and 75^th^ percentiles of the fluorescence signal across the monitored cell population over time (“Time” column in is minutes) (files: <FP_name>_mean.xlsx, <FP_name>_25.xlsx, <FP_name>_75.xlsx)
